# Changes in nasal and throat microbiota composition during and after mupirocin and chlorhexidine decolonization treatment in asymptomatic methicillin-resistant *Staphylococcus aureus* carriers: a longitudinal observational study

**DOI:** 10.1016/j.cmi.2026.02.020

**Published:** 2026-02-24

**Authors:** Sofie Marie Edslev, Cindy M. Liu, Bobby Zhao Sheng Lo, Heidi Meiniche, Berit Lilje, Daniel E. Park, Amalie Rendboe, Tony Pham, Juan E. Salazar, Tor Toudahl, Anna Cäcilia Ingham, Maliha Aziz, Mette Damkjær Bartels, Paal Skytt Andersen, Robert Leo Skov, Lance B. Price, Marc Stegger

**Affiliations:** 1) Department of Sequencing and Bioinformatics, Statens Serum Institut, Copenhagen, Denmark; 2) European Society of Clinical Microbiology and Infectious Diseases (ESCMID) Study Group for Staphylococci and Staphylococcal Diseases (ESGS), Basel, Switzerland; 3) Antibiotic Resistance Action Center, Department of Environmental and Occupational Health, Milken Institute School of Public Health, George Washington University, Washington, DC, USA; 4) Department of Clinical Microbiology and MRSA Knowledge Center, Copenhagen University Hospital – Amager and Hvidovre, Hvidovre, Denmark; 5) Department of Clinical Medicine, University of Copenhagen, Copenhagen, Denmark; 6) Antimicrobial Resistance and Infectious Diseases Laboratory, Harry Butler Institute, Murdoch University, Murdoch, Western Australia, Australia

**Keywords:** MRSA, MRSA decolonization, Mupirocin, Nasal microbiome, Throat microbiome

## Abstract

**Objectives:**

Decolonization treatment of asymptomatic methicillin-resistant *Staphylococcus aureus* (MRSA) carriage is recommended as a preventive measure in several countries, including Denmark. This study aimed to investigate the temporal dynamics of the nasal and throat microbiota in MRSA carriers undergoing decolonization treatment and to assess bacterial changes associated with treatment success.

**Methods:**

Adults with asymptomatic nasal MRSA carriage (*n* = 34) were included in this prospective observational study. Participants underwent a 5-day decolonization regimen consisting of 2% mupirocin nasal ointment and daily skin cleansing with 4% chlorhexidine soap. Nasal and throat samples were collected at multiple time points from baseline to 6 months after treatment and analysed by 16S rRNA gene sequencing. A reference group of untreated community-dwelling adults (*n* = 116) served as comparison.

**Results:**

At 1-month follow-up, 21 of 34 participants (62%) were successfully decolonized, whereas 13 (38%) remained MRSA positive at one or more body sites. At baseline, no nasal bacterial taxa were significantly associated with nasal MRSA decolonization outcome. The nasal bacterial community changed significantly within the first 2 days of treatment and remained distinct from the baseline composition for 30 days, both among those with successful decolonization and those who remained colonized with MRSA. Nasal community state types characterized by *S*. *aureus*, coagulase-negative staphylococci*, Moraxella*, and *Dolosigranulum* became less prevalent during treatment, whereas community state types dominated by *Corynebacterium* and *Cutibacterium* increased in prevalence. Among successfully decolonized participants, the nasal microbiota at 3 and 6 months after treatment continued to differ from baseline and from the reference group, mainly because of a sustained depletion of *Dolosigranulum* and *Moraxella*. In contrast, the throat microbiota showed only short-term compositional changes.

**Conclusions:**

Decolonization treatment significantly alters the nasal microbiota, with both short-term and persistent effects. Future strategies could consider supplementation with beneficial nasal commensals, such as *Dolosigranulum*, after treatment to promote microbiome recovery.

## Introduction

*Staphylococcus aureus* is an opportunistic pathogen and a common colonizer of the skin and mucous membranes, with the anterior nares and throat being preferred sites of colonization [1]. Approximately 40% of adults are colonized in the nose, with roughly half being persistent carriers [2–4]. *S. aureus* carriage is usually harmless, but is associated with increased risk of infection in certain patient groups and clinical setting (e.g. surgical procedures) [3,5,6], and decolonization treatment is therefore sometimes recommended as a preventive measure [7]. This is particularly relevant for carriers of methicillin-resistant *S. aureus* (MRSA), which represents a significant public health concern [8].

The prevalence of MRSA in Denmark is low (62 cases per 100 000 inhabitants in 2023) compared with most other European countries [9,10], because of an active anti-MRSA policy including a general recommendation by the Danish Health Authority for decolonization treatment of MRSA carriage to prevent transmission in the community and healthcare settings [11]. The recommended treatment consists of 5 days application of mupirocin nasal ointment and skin cleansing using chlorhexidine-containing soap [11]. The success rate after the first treatment is 40–65%, with MRSA throat carriage being the highest risk factor for unsuccessful treatment [12,13]. Thus, complete MRSA decolonization often requires multiple treatment rounds, underscoring the need to improve decolonization efficacy.

Although *S. aureus* is the intended target, decolonization treatment also impacts other commensal colonizers [14,15]. Key bacteria of the nasal microbiota include *Actinobacteria* (*Corynebacterium* spp. and *Cutibacterium acnes*), *Firmicutes* (*Staphylococcus* spp., *Dolosigranulum pigrum*, and *Streptococcus* spp.), and *Proteobacteria* (*Moraxella* spp. and *Haemophilus* spp.) [16–18]. Studies indicate that some nasal commensals may protect against *S. aureus* carriage. *Dolosigranulum* is inversely correlated with *S. aureus* colonization in adults [16,19–21], likely through direct inhibition of *S. aureus* [21], and some strains of coagulase-negative *Staphylococcus* (CoNS) species produce antimicrobial peptides that target *S. aureus* [22,23]. A prospective study demonstrated that mupirocin treatment not only eradicated *S. aureus* but also eliminated *Dolosigranulum* and *Moraxella nonliquefaciens* from the anterior nares [14]. However, the study only included eight *S. aureus* carriers. Given that there are at least seven distinct nasal microbiome community state types (CSTs) [18,20,24], larger studies are needed to better understand the impact of MRSA decolonization interventions on the nasal bacterial diversity.

In this study, we examine the temporal dynamics of the nasal and throat microbiota in MRSA nasal carriers undergoing decolonization treatment. The three major aims were: (i) to investigate the impact of MRSA decolonization on bacterial diversity in the nose and the throat, (ii) to assess whether bacterial taxa in the nasal microbiota at baseline are associated with successful MRSA decolonization, and (iii) to determine whether the nasal bacterial composition of MRSA carriers shifts towards that observed in a nondecolonized reference group from the general population after decolonization treatment.

## Materials and methods

### Study participants

**Fig. 1.**
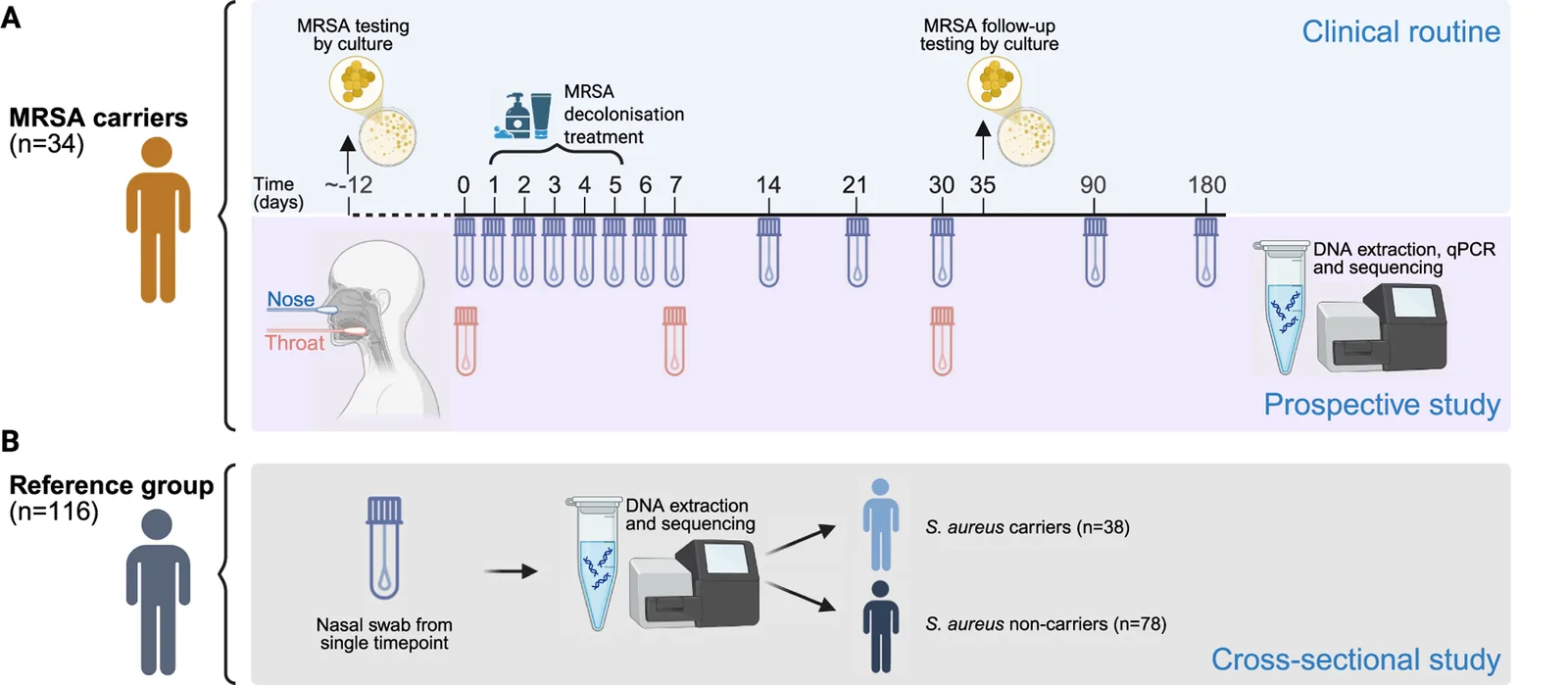
Study design. (a) MRSA carrier cohort. A total of 34 individuals with asymptomatic nasal MRSA carriage who were offered decolonization treatment by the Danish healthcare system were included. Procedures performed as part of routine clinical care (upper panel): participants tested MRSA positive in the nose a median of 12 days before study inclusion (day 0) and initiated a 5-day decolonization treatment the next day (day 1). The treatment included application of 2% mupirocin nasal ointment (three times daily) and daily skin cleansing with 4% chlorhexidine soap. Thirty days after treatment completion, participants were screened for MRSA in the nose, throat, and, when applicable, the perineum by their general practitioner. MRSA carriage was determined by culture, antimicrobial susceptibility testing, and PCR confirmation. Prospective sampling for the microbiome research study (lower panel): nasal samples for microbiome analysis were collected at 13 selected time points: at baseline (day 0, before MRSA decolonization treatment), daily from day 1 to day 7, weekly from day 7 to day 30, and at 3 and 6 months. The two late follow-up samples were included only from participants with successful decolonization (MRSA negative at all tested body sites at the 30-day clinical follow-up). Throat samples were collected at baseline, day 7, and day 30. (b) Reference group: nasal swabs from 116 community-dwelling adults were included as a reference group for compositional comparisons of the nasal microbiota. *S. aureus* nasal carriage was defined based on 16S rRNA gene sequencing data (relative abundance of *S. aureus* >0.1%). Figure created in BioRender. MRSA, methicillin-resistant *Staphylococcus aureus.*

Adults with asymptomatic MRSA nasal carriage were recruited during 2018 at the MRSA Knowledge Center (Department of Clinical Microbiology, Hvidovre Hospital, Denmark). MRSA carriage was determined by culturing, antimicrobial susceptibility testing and PCR confirmation. Exclusion criteria were age <18 years, daily contact with livestock, completion of a MRSA decolonization treatment within the past 3 months, carriage of a mupirocin-resistant MRSA, or inability to read Danish. After recruitment, participants initiated a 5-day decolonization treatment consisting of the application of 2% mupirocin nasal ointment three times daily, daily showers using 4% chlorhexidine soap, and enhanced household cleaning. Thirty days after treatment completion, participants were screened for MRSA in the nose, throat, and, when applicable, the perineum (Fig. 1).

A cross-sectional subset of a previously published cohort of healthy Danish adults from the same regional area (plasma donors at the Blood Donor Clinic at Hvidovre Hospital) was included as a reference group [18].

Ethical approval was obtained by the regional ethical committee with protocol numbers H-17021000 and H-16023435. All participants signed an informed consent.

### Sample collection

Swabs were self-collected from the anterior nares at 13 selected time points over a 6-month period, whereas throat swabs were collected by a trained healthcare worker at baseline, day 7, and day 30 (Fig. 1). At these visits, nasal swabs were also collected by the healthcare worker for paired analyses demonstrating comparable results between professional- and self-collected samples (Fig. S1). Swabs were stored in eNAT medium (Copan, Italy), designed for microbial inactivation and DNA preservation, transported to the laboratory within 7 days, and stored at −80°C until further processing. Swabs from the reference group were collected from the same nasal area and stored in eNAT medium.

### DNA extraction, qPCR, and amplicon sequencing

DNA was extracted using enzymatic and chemical lysis followed by magnetic bead-based purification (MagMax DNA Multi-Sample Ultra 2.0 Kit, Applied Biosystems, USA). Bacterial quantification and community profiling were performed on the extracted DNA by 16S rRNA gene-based broad-range qPCR [25] and amplicon sequencing (V3–V4-region). *tuf* amplicon sequencing [26] was used to resolve staphylococcal species in a subset of samples.

### Sequence processing and taxonomic classification

Cutadapt (v3.7) and DADA2 (v1.22) were used for read trimming, quality filtering, and inference of amplicon sequence variants (ASVs). 16S rRNA ASVs were taxonomic classified using the SILVA database (https://www.arb-silva.de), whereas *tuf* ASVs were classified using a dedicated *Staphylococcus* database (https://github.com/ssi-dk/staphylome/tree/master/database). 16S rRNA ASVs identified as *Staphylococcus* were classified as either *S. aureus* or CoNS by alignment of the ASV sequences against NCBI nucleotide reference database (https://blast.ncbi.nlm.nih.gov/Blast.cgi) and validated using the species-level assignment of *tuf* ASVs (Fig. S2). ASVs not classified at the order level or identified as environmental contaminants were discarded and samples with less than 2000 reads were excluded. Overall, 348 nasal and 100 throat samples from the MRSA cohort (Fig. S3) and 116 nasal samples from the reference group were included in the final analyses.

### Bioinformatics and statistics

Analyses were performed in R (v4.2.1). Microbiome analyses were conducted at genus level, with *Staphylococcus* subdivided into *S. aureus* and CoNS, except for alpha diversity, which was measured on ASV level using the Shannon index. *S. aureus* carriage was defined based on sequencing data, using a relative abundance threshold of >0.1% *S. aureus*–assigned reads within a sample.

Nasal samples were assigned to eight CSTs based on the relative abundance of key taxa (*S. aureus*, CoNS, *Dolosigranulum*, *Moraxella*, *Haemophilus*, *Corynebacterium*, *Cutibacterium*, and a mixed bacterial group [Fig. S4]), and differences in CTS distributions between groups were assessed using Fisher’s exact test. Bacterial composition was further examined using principal component analysis (PCA) visualized in biplots (FactoMineR v2.6; factoextra v1.0.7), and group-level comparisons were assessed with permutational multivariate analysis of variance using Bray–Curtis dissimilarities (vegan v2.6.4). For longitudinal analyses, permutations were restricted within subjects, and pairwise contrast tests were applied to selected time points.

Differential abundance testing was performed using maaslin2 (v1.10.0), applying linear regression models on log_2_-transformed taxa counts. Taxa were included if present with a relative abundance >1% in ≥15% of samples. Subject ID was included as a random factor in models evaluating time-related changes.

Logistic regression was used to assess associations between nasal decolonization outcome and baseline taxa colonization and abundance. Both univariate and multivariate models, including MRSA throat carriage at baseline, were conducted. Generalized linear mixed models (lme4 v1.1.30) were used to evaluate temporal changes in alpha diversity and bacterial densities, with subject ID included as a random factor. The Benjamini–Hochberg method was used to adjust p values for multiple testing.

Detailed descriptions of the study design, laboratory protocols, bioinformatics, and statistical analyses are provided in the supplementary methods.

## Results

### Patient characteristics

Thirty-four individuals with asymptomatic MRSA nasal carriage scheduled for decolonization treatment were included in the study (Fig. 1, Table 1). Six (18%) were also colonized in the throat.

At the 1-month clinical follow-up, nasal MRSA decolonization was achieved in 27 participants (79%). When considering all tested body sites (nares, throat, and perineum), 21 participants (62%) were fully decolonized, whereas 13 (38%) remained MRSA positive by culture and were therefore recommended a second treatment and excluded from the 3- and 6-month follow-ups. The decolonization rate among MRSA throat carriers was 50% (*n* = 3). The distribution of MRSA lineages stratified by decolonization outcome is shown in Table S1.

Nasal samples from 116 community-dwelling adults were included as a reference group.

### Characterization of the microbiota in the nares and throat in MRSA carriers before MRSA decolonization treatment

**Fig. 2.**
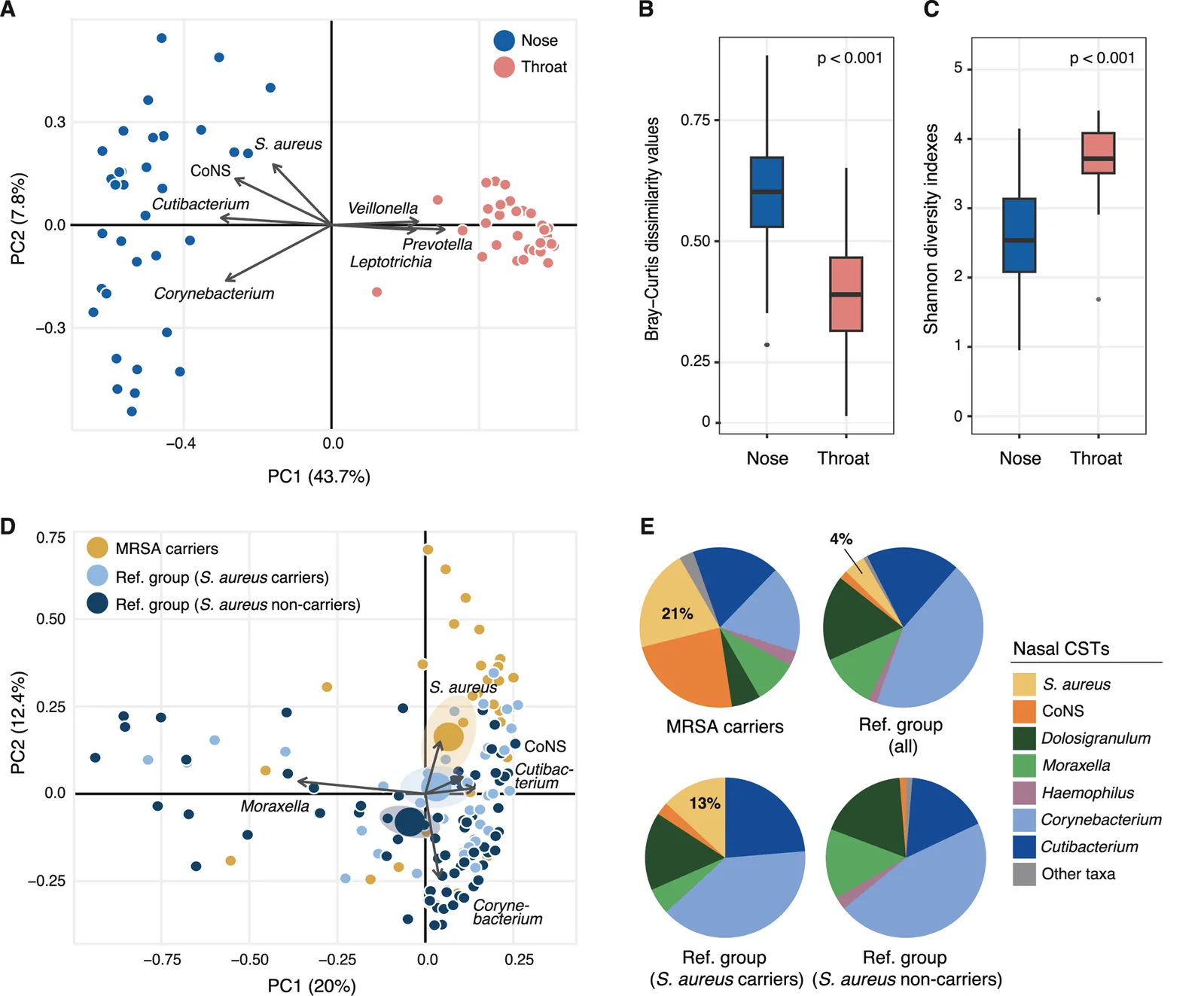
Characterization of the microbiota at baseline. (a–c) Comparison of the bacterial composition in the nose and throat of MRSA carriers. The PCA biplot (a) and box plot of Bray–Curtis dissimilarity values (interindividual pairwise comparisons for the nose and throat, respectively) (b) show greater variation in the microbiota composition between individuals in the anterior nares compared with the throat. However, alpha diversity was higher in the throat (c). (d, e) Comparison of bacterial communities in the nose of MRSA-carriers and reference population. The PCA biplot (d) shows some separation of samples collected from the MRSA carriers and the reference group, especially along PC2. The greatest distance was to the subgroup not colonized with *S. aureus*, whereas some overlap in the 95% CIs of the mean of the *S. aureus* carriers in the reference group and the MRSA carriers was observed. *Corynebacterium* and *S. aureus* were the bacterial taxa explaining most of the observed variation in PC2 (51% and 20%, respectively). The three larger points correspond to the mean point of each group with 95% CIs plotted as ellipses around the points. The pie charts (e) depict the prevalence of nasal CSTs, which were defined by key taxa in the nasal cavity, in MRSA carriers at baseline and the reference group (all participants, and stratified by *S. aureus* nasal carriage). CoNS, coagulase-negative *Staphylococcus*; CST, community state type; MRSA, methicillin-resistant *Staphylococcus aureus*; PCA, principal component analysis.

The most abundant taxa in the nose were *Corynebacterium*, *Cutibacterium*, and *Staphylococcus*, whereas *Prevotella*, *Veillonella*, and *Leptotrichia* dominated the throat microbiota (Fig. 2(a)). In addition to the compositional differences, the nasal microbiota exhibited greater interindividual variability (p < 0.001), whereas the within-sample diversity was higher in the throat (p < 0.001) (Fig.. 2(b) and (c)).

The nasal bacterial composition of MRSA carriers differed significantly from that of the reference group (adj. p 0.002), also after controlling for recent (<3 months) oral antibiotic treatment. Stratified analysis showed that the composition among *S. aureus* carriers in the reference group (*n* = 38, 33%) also differed significantly from that of MRSA carriers (adj. p 0.003), although to a lesser degree than the noncarriers (Fig. 2(d)). Grouping the nasal samples into CSTs, based on nasal indicator taxa, showed that a higher proportion of samples from MRSA carriers had a CST characterized by *S. aureus* (adj. p 0.02) or CoNS (adj. p < 0.001), whereas, the *Corynebacterium*-dominated CST was more prevalent in the reference group (adj. p 0.02) (Fig. 2(e), Table S2). No significant differences in CST distribution were observed between MRSA carriers and *S. aureus* carriers within the reference group. MRSA carriers with an *S. aureus*-CST clustered primarily in the upper right quadrant of the PCA plot (Fig. 2(d)), accounting for much of the separation from the reference group (Fig. S5).

### Baseline nasal bacterial taxa in relation to MRSA decolonization outcome

Despite being nasal MRSA carriers at the time of recruitment, *S. aureus* was not detected in the baseline nasal sequencing data from seven (21%) participants. This may reflect either methodological limitations or spontaneous clearance from the nose. Notably, six of these seven participants (86%) achieved successful decolonization at all tested body sites, supporting the hypothesis of spontaneous clearance. Two of these participants had a *Dolosigranulum*-dominated CST, comprising the full representation of this CST at baseline. However, statistical analyses showed that neither *Dolosigranulum* nor other nasal bacterial taxa at baseline were associated with the probability of a successful MRSA decolonization in the nose (Tables S3 and S4). In addition, there was no clear separation between baseline nasal samples from participant with successful and unsuccessful decolonization in the PCA plot shown in Fig. 2(d) (Fig. S5). Accordingly, MRSA carriers with an *S. aureus*–dominated CST at baseline had a nasal decolonization success rate (71%, *n* = 5) comparable with that of the overall cohort.

### Temporal dynamics of the nasal microbiota during and after MRSA decolonization treatment

**Fig. 3.**
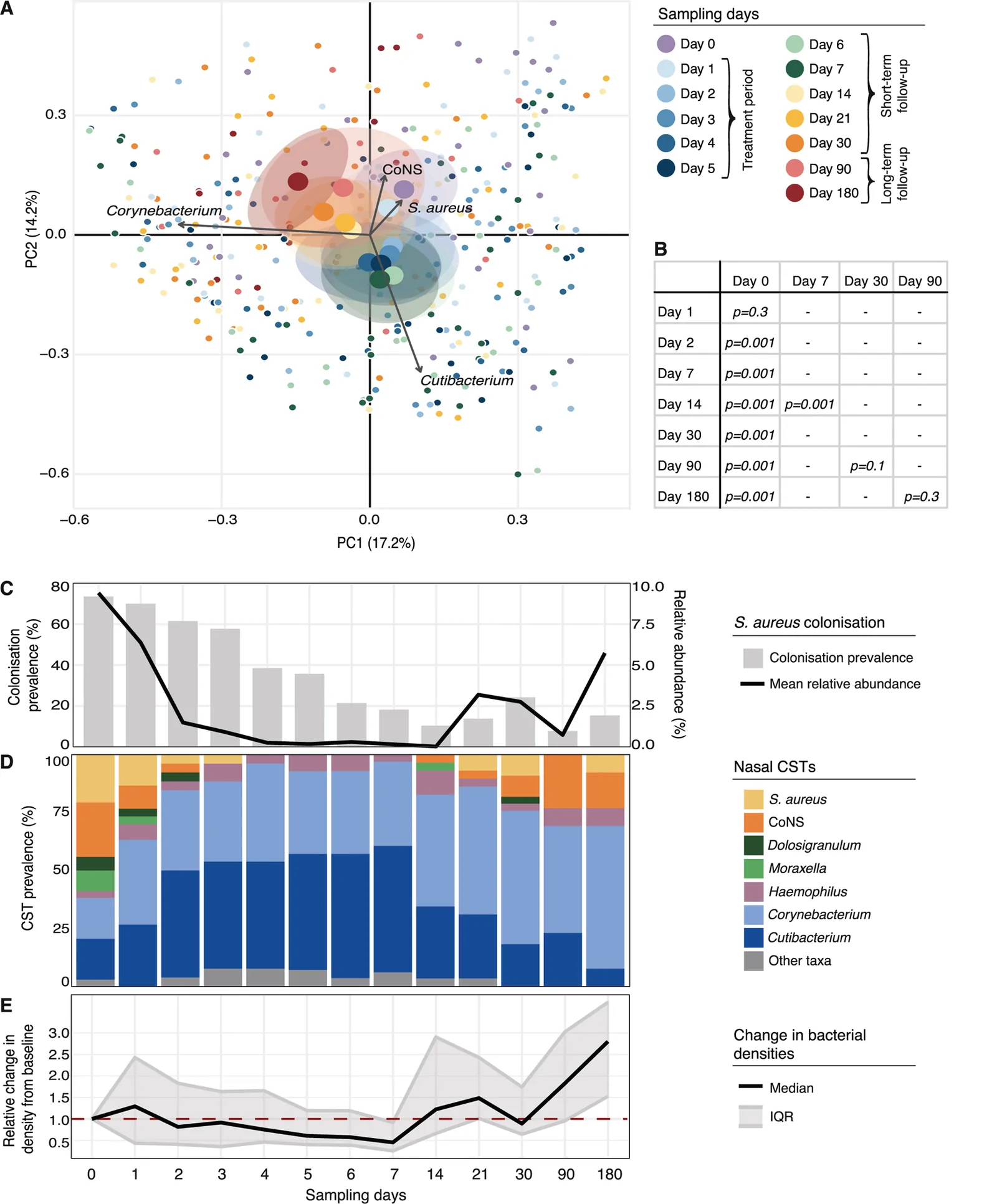
Temporal dynamics of the nasal microbiota in MRSA carriers undergoing decolonization. (a) PCA biplot illustrating temporal changes in bacterial composition during and after decolonization treatment. Larger points represent the mean for each sampling time point, with 95% CIs shown as ellipses. *Corynebacterium* and *Cutibacterium* contributed most to the variation along PC1 and PC2, respectively (66% and 63%). (b) Results from PERMANOVA pairwise contrast analyses comparing bacterial community composition between selected sampling time points. The shown p values have been adjusted for multiple testing and values < 0.05 indicate statistically significant differences in composition between time points. (c) Prevalence of participants colonised with *S. aureus* (left y-axis) and mean relative abundance of *S. aureus* (right y-axis) at each time point. The two y-axes are not equally scaled. *S. aureus* colonization was defined based on sequencing data using a relative abundance threshold of >0.1% *S. aureus*–classified reads per sample. (d) Bar plot showing the prevalence of nasal CSTs, which were defined by key taxa in the nasal cavity, across time points. (e) Temporal changes in total bacterial density, calculated for each sampling time point as the relative change from baseline, with median and IQR values shown. (c–e) All participants are included from baseline to day 30, whereas only successfully decolonized participants are included at day 90 and day 180. CoNS, coagulase-negative *Staphylococcus*; CST, community state type; IQR, interquartile range; MRSA, methicillin-resistant *Staphylococcus aureus*; PCA, principal component analysis; PERMANOVA, permutational multivariate analysis of variance.

The decolonization treatment had a significant impact on the nasal bacterial composition both in the short term and long term (Fig. 3), whereas no significant temporal changes were observed in alpha diversity (Fig. S6). A PCA biplot revealed a significant shift in community composition from baseline to the second day of treatment, which was confirmed by a permutational multivariate analysis of variance test (adj. p 0.001) (Fig.. 3(a) and (b)). This indicates an immediate effect of treatment on the nasal microbiota. The shift was characterized by a reduction in the relative abundance of *S. aureus* (log_2_ fold change = − 2.2, adj. p 0.002) and CoNS (log_2_ fold change = − 1.9, adj. p < 0.0001) (Fig. 3(c), Table S5). In the PCA biplot, samples collected from day 2 to 7 shared closely clustered centroids, suggesting a stabilization of the microbiota during the remainder of the treatment period. This was followed by a further compositional change between day 7 and 14 (adj. p 0.001), and the composition from day 14 to 180 remained significantly different from baseline (adj. p values < 0.001), indicating that in most participants, the bacterial composition did not revert to its original state. Moreover, the bacterial community structure at 1–6 months after treatment generally did not resemble that observed in the reference group (Fig .. 4(b) and (c)).

**Fig. 4.**
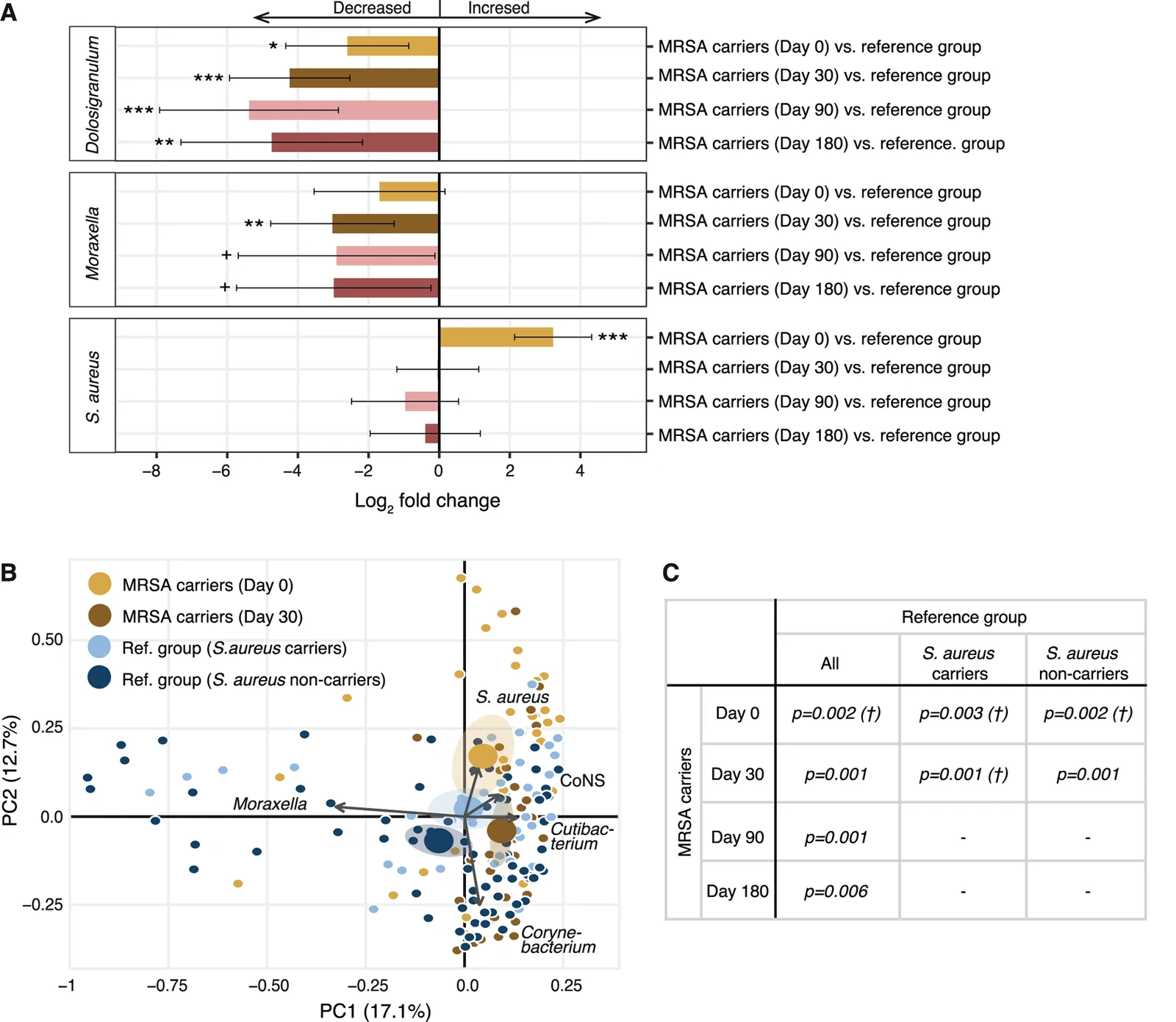
Post-treatment characterization of the nasal microbiota. (a) Results from differential abundance analyses comparing taxon abundances in MRSA carriers (four selected time points: day 0, 30, 90, and 180) with the reference group. A log_2_ fold change <0 indicates that the taxon was reduced in MRSA carriers compared with the reference group, and a log_2_ fold change >0 indicates it was enhanced. Error bars represent 95% CIs and significance levels are indicated as follows: *** adj. p < 0.001, ** adj. p < 0.01, * adj p < 0.05, and + unadjusted p < 0.05. (b) PCA biplot comparing nasal bacterial communities in MRSA carriers before (day 0) and after (day 30) decolonization treatment with those of the reference group, stratified by *S. aureus* carriage status. *Moraxella* and *Corynebacterium* contributed most to the variation along PC1 and PC2, respectively (71.7% and 56.9%). *S. aureus* was the second largest contributor to PC2 (16.8%). (c) Results from PERMANOVA pairwise analyses comparing bacterial community composition between selected groups. p values are adjusted for multiple testing and values < 0.05 indicate statistically significant differences in composition between time points. ^†^ indicates unequal dispersion of samples between the tested groups. CoNS, coagulase-negative *Staphylococcus*; *MRSA, methicillin-resistant Staphylococcus aureus;* PCA, principal component analysis; PERMANOVA, permutational multivariate analysis of variance.

The observed compositional changes were also reflected in the temporal prevalence of CSTs (Fig. 3(d) and S7). Specifically, CSTs characterized by *S. aureus*, CoNS*, Moraxella*, and *Dolosigranulum* became less prevalent during treatment, whereas CSTs dominated by *Corynebacterium* and *Cutibacterium* became more prevalent, consistent with the known lack of mupirocin effect against these taxa [15]. Although *S. aureus* and CoNS CSTs later rebounded, the *Dolosigranulum* and *Moraxella* CSTs remained sparse. Abundances of *Dolosigranulum* and *Moraxella* were also significantly reduced in MRSA-carriers after treatment compared with the reference group (Fig. 4(a), Table S6).

In addition to the compositional shifts, decolonization treatment also affected the total bacterial density in the nose (p 0.01). A slight decrease in the median density was observed between day 2 and 7, after which it began to rise, culminating in a more than two-fold increase at day 180 compared with baseline (Fig. 3(e)).

At the day 30 sampling, which coincides with routine clinical follow-up screening for MRSA, no significant differences in overall community structure were observed between participants with successful and unsuccessful MRSA decolonization (p 0.7) (Fig. S8). Although *S. aureus* abundance was higher in participants with persistent nasal MRSA carriage (log_2_ fold change = 3.5, p 0.01), this difference was insignificant after adjustment for multiple testing, and no other taxa differed significantly between the groups.

### Temporal dynamics of the throat microbiota during and after MRSA decolonization treatment

Prospective analysis of the throat microbiota indicated that decolonization treatment had a short-term (day 7 vs. baseline, adj. p 0.002), but not a long-term (day 30 vs. baseline, adj. p 0.2), effect on the bacterial composition (Fig. S9, Table S7). Alpha diversity and the bacterial density did not change over time in the throat.

## Discussion

We observed that standard MRSA decolonization treatment had a sustained impact on the nasal bacterial composition and density, whereas no long-term effects were observed in the throat microbiota.

As expected, the nasal abundance of *S. aureus,* CoNS, and *Moraxella* declined during treatment, consistent with the known inhibitory activity of mupirocin against these taxa [15]. Our findings further suggest that mupirocin also inhibit *Dolosigranulum*, a finding consistent with previous observations [14]. Importantly, these treatment-related changes persisted beyond the treatment window. Thus, *Dolosigranulum* and *Moraxella* were only sparsely re-established in the months after treatment cessation and remained significantly underrepresented as compared with the reference group. This persistent depletion is unintended, as *Dolosigranulum* likely has a protective role against *S. aureus* colonization [19–21]. In accordance, this study may suggest that *Dolosigranulum* nasal colonization could be associated with spontaneous MRSA decolonization, as *S. aureus* was not detected in baseline samples from the two participants with a *Dolosigranulum*-dominated CST. However, this observation requires confirmation in larger, independent studies.

A significant increase in nasal bacterial density was observed 6 months after decolonization, consistent with previous findings [27]. This may reflect altered ecological dynamics after eradication of specific taxa, allowing other bacteria to establish, a notion supported by the observation that the microbiota composition did not revert to baseline 3–6 months after treatment.

None of the nasal taxa present at baseline were significantly associated with nasal MRSA decolonization outcome, suggesting that other factors, such as hygiene measures, MRSA colonization at other body sites or exposure from close contacts [28], may have a stronger influence on treatment outcome. Moreover, because mupirocin also targets *Dolosigranulum* and CoNS, any potential beneficial effects of these taxa may have been reduced. An interesting future direction would be to investigate whether supplementation with beneficial commensal strains, such as *Dolosigranulum* or certain CoNS, administered intranasally after decolonization could promote the establishment of a resilient microbiota resistant to MRSA recolonization.

A recent study reported that carriage of an *S. aureus*-dominated nasal CST was significantly associated with persistent *S. aureus* carriage [24]. In this study, participants with an *S. aureus* CST at baseline contributed substantially to the compositional differences between MRSA carriers and the reference group. However, our findings do not indicate reduced decolonization success among MRSA carriers with an *S. aureus* CST.

The main limitation of this study is the modest cohort size, which may limit statistical power to detect associations between specific taxa and MRSA decolonization outcomes. However, the intensive sampling strategy provides a unique dataset for investigating bacterial dynamics related to decolonization treatment, a topic that remains underexplored in current literature [14,27]. A second limitation is the lack prospective sampling and antibiotic susceptibility data for *S. aureus* in the reference group; however, given the low MRSA prevalence in Denmark [9], it is unlikely that they were colonized with MRSA.

In conclusion, this study demonstrates that MRSA decolonization treatment has a measurable impact on the nasal microbiota, with effects observed both immediately and over the longer term. These findings underscore the importance of accounting for microbiome effects when developing decolonization treatment strategies.

## Supplementary Material

1

2

## Figures and Tables

**Table 1:** Description of the study participants

Variable	MRSA carriers (*n* = 34)	Reference group (*N* = 116)	p	MRSA carriers w. successful decolonization (*n* = 21)	MRSA carriers w. unsuccessful decolonization (*n* = 13)	p

Sex (F/M)	59%/41%	50%/50%	0.4	52%/48%	69%/30%	0.5
Age (y), median (range)	45 (25–80)	47 (25–66)	0.7	39 (25–75)	53 (29–80)	0.2
Smoking	4 (12%)	13 (11%)	1	3 (14%)	1 (8%)	1
MRSA throat carriage^a^	6 (18%)	na		3 (14%)	3 (23%)	0.6
Recent MRSA infection^b^	13 (37%)	na		9 (43%)	4 (31%)	0.5
Recently treated, oral antibiotics^c^	17 (50%)	10 (9%)	<0.001	11 (52%)	6 (46%)	0.7
Recently treated, topical antibiotics^d^	9 (27%)	0	1	3 (14%)	6 (46%)	0.1

Pairwise comparison of groups (MRSA carriers vs. reference group; and MRSA carriers with successful decolonization vs. unsuccessful decolonization) were performed using Fisher’s exact test. Decolonization outcome was assessed by culturing of swabs from the nose, throat, and perineum, with an MRSA-positive sample from any tested body site indicating unsuccessful decolonization. na, not available.

a Missing information from two of the MRSA-carriers.

b Previous MRSA infection within the last 3 months before treatment initiation, missing information from one of the MRSA carriers.

c,d Treated with oral administered antibiotics (systemic) or topical administered antibiotics within the last 3 months, missing information from one of the MRSA carriers.

## Data Availability

Sequence data from the cohort of MRSA-carriers are available at the European Nucleotide Archive (ENA) under the BioProject ID PRJEB98419, and sequence data from the reference group are available from the NIH Sequence Read Archive (SRA) under PRJNA1249528. Other data can become available for research upon requests to the corresponding author, and within the framework of the Danish data protection legislation and any required permission from Authorities.

## Appendix A. Supplementary data

Supplementary data to this article can be found online at https://doi.org/10.1016/j.cmi.2026.02.020.
