# Supplementary material for: Changes in nasal and throat microbiota composition during and after mupirocin and chlorhexidine decolonization treatment in asymptomatic methicillin-resistant Staphylococcus aureus carriers: a longitudinal observational study
Source: Clin Microbiol Infect. Author manuscript; Available in PMC 2026 Sep 12. (PMC13570456; doi:10.1016/j.cmi.2026.02.020)
Supplement: Supplementary file 1 [file NIHMS2190782-supplement-1.pdf]

Supplementary methodology

Clinical practice for MRSA diagnostics in Denmark

MRSA nasal carriage was defined based on a single positive screening swab from the nose. In addition to the nasal swab, a swab from the throat and, often but not always, a sample from the perineum are collected to screen for MRSA carriage at these other common colonization sites.

Screening swabs were prior to cultivation on chromogenic MRSA plates and non-selective 5% horse blood agar inoculated into an MRSA tryptic soy broth containing 2.5% salt, 3.5 mg/L cefoxitin, and 20 mg/L aztreonam and incubated overnight. MALDI-TOF (Bruker) was used to confirm growth of *S. aureus*. EUCAST cefoxitin disk diffusion breakpoints were used for phenotypical confirmation of MRSA and a colony from a pure culture was sent to an in-house PCR with targets for the genes *nuc*, *femA*, *mecA*, and *mecC* for final confirmation of MRSA (1).

Recruitment procedure and sample collection from MRSA carriers

Medical personal at the MRSA Knowledge Center, Department of Clinical Microbiology at Hvidovre Hospital (Copenhagen, Denmark), were responsible for the recruitment of participants. Lists of MRSA-positive screening samples, generated daily from the Laboratory Information System, were reviewed to identify patients who met the inclusion criteria. The inclusion criteria were age 18 or older and MRSA in the nose that was susceptible to mupirocin. Patients that had previously undergone MRSA decolonization treatment including mupirocin, had the livestock associated CC398 clone/were suspected to have CC398 due to daily contact with livestock, or did not to speak or read Danish were excluded. Positive screening sites other than nose (throat and perineum) was not an exclusion criterion.

Eligible patients were contacted by telephone by one of the doctors, who provided verbal information about the project. If the patient expressed interest in participation, written study information was sent by email. Patients were asked to respond within five days and were encouraged to contact the MRSA team by phone or email if they had any questions about the study. Patients who agreed to participate signed an informed consent form.

One day prior to treatment initiation, a trained medical professional conducted a home visit. During the visit, participants were instructed on how to perform nasal self-sampling correctly. The medical

staff also collected baseline nasal and throat swabs. Nasal swabs were taken from both nostrils using the same swab, which was inserted approximately 1 cm into the nasal cavity and rotated three times against the mucosal surface. Throat swabs were collected by pressing the swab against both tonsils (or the tonsillar area) for a few seconds. Baseline samples were used exclusively for DNA-based microbiome analyses and were not cultured to reconfirm MRSA carriage.

Participants completed a questionnaire during the first visit, which included questions about occupation, smoking status, allergies, diabetes, asthma, MRSA infections, antibiotic use within the past three months, and treatment for allergies or asthma.

The time interval between a positive MRSA screening result and the first study visit had a median of 12 days (interquartile range: 8–20 days), with an overall range of 4–111 days. Participants were instructed to self-collect nasal swabs daily during the first week after treatment initiation and to label the samples accordingly. Swabs were stored in eNAT medium (Copan, Italy), designed for microbial inactivation and preservation of microbial DNA. This allowed samples to be stored at room temperature until day 7, when the healthcare worker conducted the second visit. During this visit, new nasal and throat swabs were collected, and all previously collected samples were picked up and transported to the Department of Clinical Microbiology at Hvidovre Hospital, where they were stored at –80°C.

Participants also self-collected nasal swabs on day 14 and day 21, which were collected by the healthcare worker during a third home visit, 30 days after treatment initiation, and stored at –80°C at the Department of Clinical Microbiology at Hvidovre Hospita.

Throat swabs were collected only during visits by a trained medical professional, as repeated self-collection from the throat was considered impractical and potentially uncomfortable for participants.

Thirty days after treatment completion, participants attended a routine follow-up visit with their general practitioner, as per the Danish National MRSA Guidelines. At this visit, swabs were collected from the nose, throat, and, in some cases, the perineum. Samples were sent for culture and antimicrobial susceptibility testing to determine MRSA colonization status. Doctors at the MRSA Knowledge Center reviewed test results in the Laboratory Information System. Participants who tested negative for MRSA at all tested body sites were defined as successfully decolonized and were included in the 3–6-month follow-up phase. Participants who remained MRSA-positive were excluded from further follow-up.

For those included in the follow-up study, sampling kits were mailed to participants approximately one week prior to the scheduled sampling date. The kits included instructions and a prepaid return envelope for mailing the samples back to the laboratory.

MRSA decolonization treatment procedure

All participants received both oral and written information about the treatment procedure in accordance with the national MRSA guidelines. The written information, translated from Danish to English, is provided below.

Treatment Instructions

The treatment lasts for 5 days and includes elimination of the bacteria from:

1. The nose, using a special antibiotic nasal ointment
2. The skin and hair, by washing with an antibacterial soap
3. The home, through cleaning and laundry routines

You and all members of the household must follow the procedure below carefully.

1. Elimination of bacteria from the nose

- Apply nasal ointment (*Mupirocin 2%*) to both nostrils three times daily. Use a separate cotton swab for each nostril. Only apply inside the visible part of the nostrils — not deeper than a finger can reach. Use an amount of ointment equivalent to the size of a pea for each nostril. The entire tube should be used over the 5-day treatment period.
- After application, gently press the nostrils together to distribute the ointment.
- Wash hands afterward using chlorhexidine soap 4%.

2. Elimination of bacteria from skin and hair

Avoid wearing jewelry during treatment, especially rings, earrings, and body piercings. You may continue to use conditioner, deodorant, aftershave, and similar products, but do not use any soap or shampoo other than chlorhexidine soap.

Take a shower (not a bath) daily, washing both body and hair with chlorhexidine soap 4%. Pay special attention to the nose, mouth, armpits, navel, genitals, and groin. If using reusable washcloths, boil-wash them. Single-use cloths must be discarded after use.

To prevent skin dryness, you may apply moisturizer after washing. However, some creams may reduce the effect of chlorhexidine, so only use moisturizers listed in the attached approved product list.

If the treatment causes rash or significant skin irritation, contact your physician. It is recommended to replace your toothbrush during the treatment, especially if you are colonized with MRSA in the throat. Dispose of the old toothbrush.

3. Elimination of bacteria from the home

- Each household member should use their own towels and washcloths. Change into clean underwear, towels, and washcloths daily after showering with chlorhexidine, as described in the attached washing instructions.
- All household members should change to clean bed linen on day 2 and day 5 of treatment.
- Bed linen, towels, washcloths, underwear, dishcloths, and kitchen towels must be boil-washed after use. If possible, duvets and pillows should also be washed at the end of the treatment.
- Air out the home daily, including airing out duvets and pillows.
- Clean all horizontal surfaces with water and soap.
- Vacuum the home on day 2 and day 5. Mattresses and upholstered furniture must also be vacuumed thoroughly.
- Frequently touched surfaces such as door handles, mobile phones, faucets, toilet seats, flush buttons, and car steering wheels must be cleaned daily.

Description of the reference group

A subset of nasal samples from a previously published (2) cohort of community-dwelling adults (plasma donors at Hvidovre Hospital, Copenhagen, Denmark) were included in the study. The subset was selected to ensure that participants were matched by age and sex to the MRSA-carrier cohort,

and that samples were collected throughout the year to ensure seasonal representation. Taxonomic composition was not part of the selection criteria.

Nasal samples were collected from the same anatomical area of the nose and stored in the same transport medium (eNAT) as those collected from the MRSA carrier cohort.

Samples from the reference group were used exclusively for DNA-based sequencing (16S rRNA amplicon sequencing) and were not cultured. Accordingly, *S. aureus* carriage in this cohort was defined based on sequencing data rather than culture results. Specifically, the presence of *S. aureus*-classified reads with a relative abundance >0.1% within a sample was used to define *S. aureus* carriage in the reference group. As samples from the reference group were not cultured, it was not possible to determine whether *S. aureus* carriers in this group were colonised with MRSA or MSSA; however, given the low prevalence of MRSA in Denmark, MRSA carriage in the reference group is considered unlikely.

DNA extraction

DNA was extracted from nasal swab samples using the MagMAX Microbiome Ultra Nucleic Acid Isolation Kit (Thermo Fisher Scientific, MA, USA) following a modified version of the manufacturer's protocol. Prior to extraction, samples (80 µL of the storage medium) were enzymatically pre-treated to improve lysis of Gram-positive bacteria. The pre-treatment consisted of incubation with a lysis buffer containing 5 µL lysozyme (100mg/mL solution, Sigma), 4 µL mutanolysin (25 KU/mL, Sigma), and 3 µL lysostaphin (4 U/µL, Sigma) at 37 °C for 1 hour. After enzymatic treatment, samples were incubated with 25.9 µL Proteinase K for at least 20 minutes at 65 °C. DNA extraction was conducted on a Hamilton STAR robot suing the MagMAX DNA Multi-Sample 2.0 Kit (Applied Biosystems by Thermo Fisher Scientific). Following incubation, 440 uL DNA Lysis/Binding Bead Mix was added to each sample. Wash steps included a series of ethanol-based washes and magnetic bead separation to remove inhibitors and contaminants. DNA was eluted in 80 µL of elution buffer and stored at –20°C until further analysis. Throughout the protocol, negative extraction controls and positive extraction controls were included to monitor for contamination and quality measurements.

DNA quantification for each sample was performed by pan bacterial qPCR. Concentration and purity

of DNA libraries for downstream 16S amplicon sequencing were assessed using a Qubit fluorometer and Bioanalyzer.

16S rRNA amplicon sequencing

The V3-V4 region of the 16S rRNA gene was amplified using the primers from Fadrosh *et al*. (3) in a 25 cycle PCR (3 min. initial denaturation at 98°C, 25 cycles of 30s at 98 °C, 30s at 58 °C, and 60s at 72°C, followed by a final extension for 7 min. at 72°C.). Next, samples were indexed in a second PCR using Ilumina index primers (3 min. initial denaturation at 98 °C, 8 cycles of 30s at 98 °C, 30s at 55°C, and 30s at 72°C, followed by a final extension for 5 min. at 72°C). The DNA concentrations were quantified using AccuClear ultra high sensitivity kit (Biotium, CA, USA) and samples were normalized and pooled followed by an AMPure XP bead clean-up (x1.12 bead ratio). Amplicon libraries were sequenced on a MiSeq instrument using V3 reagent kits (600 cycles) and 15% phiX (Illumina Inc., San Diego, CA, USA).

qPCR targeting the 16S rRNA gene

Bacterial quantification was performed using a 16S rRNA gene-based broad-range qPCR (BactQuant) (4). All assays were performed in 10 µL reactions consisting of 1 µL of template DNA and 9 µL of qPCR reaction mix containing 1x QuantaBio PerfeCTa qPCR ToughMix with ROX, DMSO, 0.90 µM forward primer, 0.90 µM reverse primer, 0.25 µM Applied Biosystems TaqMan MGB probe, and molecular grade water. Thermal cycling conditions were as follows: initial denaturation for 3 minutes at 95°C, followed by 40 cycles of denaturation (15 seconds at 95°C) and annealing/extension (60 seconds at 60°C). All assays are performed on Roche LightCycler 480-II (Roche 05015243001).

tuf amplicon sequencing

To further resolve staphylococcal species within complex communities, *tuf* gene amplicon sequencing (5) was carried out on a subset of samples. The *tuf* gene was amplified using the following primers deigned with heterogeneity spacers:

F1: 5’CAGAAGAAAAAGAACGTGG-3’

F2: 5'TCAGAAGAAAAAGAACGTGG-3'

F3: 5'-GTCAGAAGAAAAAGAACGTGG-3'

F4:5'AGTCTCTCGTCAGAAGAAAAAGAACGTGG-3

R1: 5'-GTCCTCAACWGGCATCA-3'

R2: 5'-AGTCCTCAACWGGCATCA-3'

R3: 5'-CAGTCCTCAACWGGCATCA-3'

R4: 5'-TCAGGTCCTCAACWGGCATCA-3'

The selected gene region were initially amplified in a 25 cycle PCR (3 min. initial denaturation at 95°C, 25 cycles of 20s at 98°C, 15s at 60°C, and 45s at 72°C, followed by a final extension for 5 min. at 72 °C.). Next, samples were indexed in a second PCR using Ilumina index primers (3 min. initial denaturation at 95°C, 20 cycles of 20s at 98°C, 15s at 55°C, and 45s at 72°C, followed by a final extenstion for 5 min. at 72°C). Primer and primer dimer products were removed using AMPure XP (Beckman Coulter, Inc., CA, USA) magnetic bead clean-up (x0.72 bead ratio). The DNA concentrations were quantified using AccuClear ultra high sensitivity kit (Biotium, CA, USA) and samples were normalized and pooled followed by an additional AMPure XP bead clean-up (x0.8 bead ratio). Amplicon libraries were sequenced on a MiSeq instrument using V3 reagent kits (600 cycles) and 5% phiX (Illumina Inc., San Diego, CA, USA).

Sequence preprocessing and taxonomic classification

Cutadapt v3.7 (6) was used for primer trimming at an 8% error rate, corresponding to one mismatch per primer, in paired-end mode. Run-wise sequence quality filtering was performed with DADA2 v1.22 (7) using default settings, with the exception of truncation length. I.e., 16S rRNA amplicon reads were truncated at 270 bp (forward reads) and 210 bp (reverse reads), and *tuf* amplicon reads were truncated at 270 bp and 241 bp. Chimera filtering was performed on all sequencing runs combined.

DADA2 v1.22 was used for construction of amplicon sequence variants (ASVs) and taxonomic assignments. The 16S rRNA derived ASVs were classified using the assignTaxonomy and addSpecies functions, using the reference database Silva v132.1 and the species-level training set

formatted for DADA2 (8). The *tuf* derived ASVs were classified using the assignTaxonomy function and an inhouse created staphylococcal-specific taxonomy database, which can be found her: https://github.com/ssi-dk/staphylome/tree/master/database.

All 16S rRNA ASVs identified as *Staphylococcus* were classified as either *S. aureus* or coagulase negative staphylococci (CoNS) by BLAST'ing the eight most abundant staphylococcal ASV sequences against the nucleotide reference database managed by the National Center for Biotechnology Information (NCBI) (https://blast.ncbi.nlm.nih.gov/Blast.cgi). *Tuf* sequencing was performed on a subset of samples (n=31) to validate the species-level classification of staphylococcal ASVs. Spearman correlation tests were conducted to assess the relationship between the relative abundance of each of the eight dominant staphylococcal ASVs (derived from the 16S rRNA dataset) and the five most abundant staphylococcal species identified in the *tuf* dataset, within the same samples. Taxonomic bar plots were also generated for manual inspection of ASV–species distribution in the paired samples. These analyses confirmed the species assignments of the eight selected ASVs, with one corresponding to *S. aureus* and the remaining seven to CoNS. Next, a SNP-based phylogeny was constructed using all staphylococcal ASVs (n=202) extracted from the 16S rRNA dataset to evaluate their sequence similarity to the eight validated ASVs. This pairwise comparison enabled the assignment of 188 out of the remaining 194 ASVs to either *S. aureus* or CoNS.

Sequence data quality check

ASV and taxonomy tables were merged with sample metadata into a *phyloseq* object in R using the R package phyloseq v.1.40.0 (9). Potential batch effects across sequencing runs were assessed using principal coordinates analysis (PCoA), which revealed no systematic differences between sequencing batches.

Contamination was evaluated by inspecting taxonomic profiles of both negative controls and positive controls (mock communities). ASVs assigned to known environmentally derived contaminant phyla (Cyanobacteria, Planctomycetota, Chloroflexi, Patescibacteria, Nitrospirota, Aquificota, Desulfobacterota, Methylomirabilota, Elusimicrobiota, and Dadabacteria) were removed. Additionally, ASVs not classified at least at the order level, as well as those classified as Archaea, were excluded.

Sequencing depth was assessed using rarefaction curves, which showed that most samples plateaued after ~2,000 reads. This informed the decision to use 2,000 reads as the minimum cutoff for downstream analysis. To validate this threshold, ASV richness was compared across different inclusion thresholds (>2,000, >2,500, and >3,000 reads) using a Kruskal–Wallis test, which revealed no significant differences. Samples with fewer than 2,000 reads (n=33) were excluded.

To evaluate the quality of self-sampling, paired nasal samples collected by participants and the trained medical staff at the same time point were compared (n=2×85). First, bar plots of the 25 most abundant taxa were visually inspected. Second, Bray–Curtis dissimilarities were calculated and hierarchical clustering (Ward D method) was performed, showing that paired samples clustered closely. Third, sequencing depth was compared between groups using a paired Wilcoxon signed-rank test. Combined, these results indicated that nasal microbiome samples collected by participants were comparable in quality to those collected by trained professionals. For downstream analyses, one of each sample pair was retained (the sample with the highest sequence count).

Statistics

Statistical analyses and visualizations were performed in R (v4.2.1) (R Core Team, 2022) using phyloseq (v1.40.0) (9) for data management and ggplot2 (v3.4.1) (10) for visualizations.

Fisher's exact test was used to assess differences between groups (MRSA carriers vs. reference group as well as MRSA carriers with a successful vs. unsuccessful decolonization treatment) to evaluate potential confounding. All microbiome analyses, except for alpha diversity, were conducted at genus level, with *Staphylococcus* divided in to *S. aureus* and CoNS.

Nasal samples were assigned to eight community state types (CSTs) based on the relative abundance of the following key nasal taxa: *S. aureus*, CoNS, *Dolosigranulum*, *Moraxella*, *Haemophilus*, *Corynebacterium*, and *Cutibacterium*, and a mixed bacterial group. These community groups were defined *a priori* using the following ordered criteria: 1) samples with a relative abundance of *S. aureus* > 10% were assigned to the *S. aureus* group; 2) samples with a relative abundance of *Dolosigranulum* >10% were assigned to *Dolosigranulum* group, 3) samples with a relative abundance of *Haemophilus* >10% were assigned to *Haemophilus* group , 4) samples with a relative abundance >10% of CoNS, *Moraxella*, *Corynebacterium*, and/or *Cutibacterium* were assigned to the corresponding group and in cases where the proportional abundance were > 10% for several of these taxa, the assignment was

based on the taxa with the highest relative abundance. In a few samples, none of these taxa were present at higher abundance (>10%), and these samples were grouped as "other taxa."

Analysis of baseline samples

A principal component analysis (PCA) was conducted using the *PCA()* function implemented in FactoMineR (v2.6) (11) to compare the bacterial composition of nasal cavity and throat samples collected at baseline, where taxa counts were Hellinger-transformed prior to analysis. The results were visualized using the *fviz_pca_biplot()* function implemented in the factoextra (v1.0.7) package (12), with the seven taxa contributing most to the observed variation within PC1 and PC2 were plotted as loading vectors in the biplot. The top contributing variables (i.e. taxa) was calculated using the standard setting in the *PCA()* function. Differences between nasal and throat microbiota were further assessed by comparing between-person variability, measured by Bray–Curtis dissimilarities, and within-person diversity, measured by the Shannon index. The Shannon index was calculated based on untransformed ASV counts. An unpaired Mann–Whitney U test and a paired Wilcoxon signed-rank test were used to assess the statistical significance of differences between sampling sites in the respective analyses.

PCA-biplots were also created to compare the nasal bacterial composition at baseline with the composition observed in the reference group, stratified based on *S. aureus* carriage, which was defined as the presence of ASVs assigned to *S. aureus*. Thus, not by culturing. The five taxa contributing most to the observed variation within PC1 and PC2 were plotted as loading vectors in the biplot. Differences between the groups (MRSA carriers at baseline vs. total reference group and MRSA carriers at baseline vs. *S. aureus* carriers in the reference group) were assessed by permutational multivariate analysis of variance (PERMANOVA) using Bray-Curtis dissimilarities (*adonis2()* and *dist()* functions implemented in vegan, v2.6.4, (13)). The Bray-Curtis dissimilarities were calculated on Hellinger transformed taxa counts. Both a univariate analysis and multivariate analysis including treatments with oral antibiotics in the past three months (yes/no) as a covariate in the model was performed. Homogeneity of multivariate dispersions variances was tested using the *betadisper()* function in vegan.

The Prevalence of nasal CSTs within the MRSA carriers and reference group (all, and stratified by *S. aureus* carriage) was visualized in circle diagrams, and a Fishers exact test was used to test for differences between groups.

Microbiome Multivariable Association with Linear Models, MaAsLin2 (v1.10.0) (14), was used for testing if any taxa were enriched or reduced in the MRSA carriers compared to the reference group. A linear regression model (analysis_method = "LM") on log_2-transformed taxa counts (transform = "LOG") were applied. Taxa were included if present with a relative abundance >1% in ≥15% of samples, and p-values adjusted for multiple testing using the Benjamini-Hochberg method (correction = "BH").

PCA and PERMANOVA were also used to investigate if MRSA throat carriage at baseline had an impact on the nasal bacterial composition at baseline.

Analysis of baseline nasal taxa predicting MRSA nasal decolonization

In this sub-analysis, we investigated whether the presence and/or abundance of nasal taxa at baseline could predict MRSA decolonization of the nose. In this analysis, decolonization was defined solely based on MRSA test results from the nose, excluding throat and perineum results.

Logistic regression was used to assess associations between nasal decolonization outcome (dependent variable) and taxon presence or abundance at baseline (independent variables). Both colonization status (defined as a relative abundance >0.1%) and relative abundance (%) were evaluated in univariate and multivariate models including throat MRSA carriage at baseline as a covariate. For colonization status models, only taxa present in more than 15% and fewer than 85% of samples were tested. For abundance models, taxa with a relative abundance >1% in at least 15% of samples were tested. P-values were adjusted for multiple testing using the Benjamini–Hochberg method.

Temporal dynamics of the nasal microbiome

A PCA biplot was generated to visualize temporal compositional changes in the nasal microbiota throughout the study period, using the same procedure as described above, except for the plotting of loading vectors. In this case, the list of contributing taxa was filtered to include only key nasal taxa used to define the bacterial community groups, and the four taxa with the highest contributions to PC1 and PC2 were plotted. Contributions were calculated using the standard method described above.

Sampling time was used as the independent variable in a PERMANOVA test to investigate whether decolonization treatment was associated with compositional changes over time. Permutations were restricted within subjects to account for repeated measures. Pairwise differences between selected time points were tested using contrast analyses, where each test compared two time points while adjusting for variation captured by the full model including all samples. P-values from the contrast analyses were adjusted for multiple testing using the Benjamini–Hochberg method.

Differential abundance analysis was conducted to test which taxa that drew the compositional changes observed between baseline and day 2 as well as between baseline and day 30. MaAsLin2 was applied using the same setting as described above. The first model included nasal samples from day 0 and day 2, and the second model included nasal samples from day 0 and day 30. In both models, sampling time (factor) was set as the fixed effect and subject ID as a random effect, due to repeated measurement.
To illustrate temporal changes in *S. aureus* carriage and abundance, a plot was generated showing changes in colonisation prevalence over time, as well as changes in mean relative abundance of *S. aureus* across sampling time points. Colonisation was here defined as the presence of *S. aureus* assigned reads with a relative abundance >0.1%.

A PCA biplot, PERMANOVA, and differential abundance test were conducted to assess whether the bacterial community at day 30 differed between participants with successful versus unsuccessful decolonization. Treatment outcome was defined based on routine follow-up in the healthcare system; specifically, successful decolonization was defined as testing MRSA-negative 30 days post-treatment in both the nares and throat (and perineum, if tested). The PCA, PERMANOVA, and differential abundance analyses were performed using the same settings as described for the baseline analysis, but with treatment outcome as the independent variable. Since all samples were collected at the same time point (day 30), there was no need to adjust for subject ID.

Similarly, a PCA biplot, PERMANOVA, and differential abundance testing were conducted to assess whether the microbiome shifted toward a composition more similar to that of the reference group. In some of the analyses, the reference group was divided into two subgroups based on *S. aureus* carriage status, as determined by sequencing. The PCA biplot included samples from four groups: MRSA carriers at baseline (day 0), MRSA carriers post-treatment (day 30), *S. aureus* carriers in the reference group, and non-carriers in the reference group. The five taxa contributing most to the variation along

PC1 and PC2 were plotted as loading vectors. Multiple univariate PERMANOVA tests were performed to assess pairwise differences in bacterial community composition between the reference group (all, *S. aureus* carriers, and non-carriers) and MRSA carriers (day 0, day 30, day 90, and day 180). P-values were adjusted for multiple testing using the Benjamini–Hochberg method. Similarly, multiple MaAsLin2 models were used to test for pairwise differences in taxon abundance between the reference group and MRSA carriers at each of the four time points. Bar plots were generated to visualize the log fold change in abundance of *Dolosigranulum* and *Moraxella* between the reference group and MRSA carriers at each time point, including 95% confidence intervals as error bars and asterisks indicating significance levels.

Stacked bar plots were created to visualize changes in the prevalence of nasal CSTs over time and a dot plot visualized patient-wise changes in CST assignment.

Temporal changes in alpha diversity were assessed by calculating the Shannon index using untransformed ASV counts, followed by statistical testing using a generalized linear mixed model (*lmer()* function, lme4 v1.1.30, (15)), with sampling time included as a fixed effect and subject ID as a random effect.

Changes in bacterial density, measured by qPCR, were calculated as the difference between baseline and each follow-up time point. The relative change from baseline over time was visualized by plotting the median change along with the interquartile range (IQR1 and IQR3). A generalized linear mixed model was used to test for statistical significance, with sampling time included as a fixed effect and subject ID as a random effect, as described above.

<u>Temporal dynamics of the throat microbiome</u>

Temporal changes in the throat microbiome were investigated using PCA biplots and PERMANOVA to assess beta diversity, MaAsLin2 to identify differentially abundant taxa, and a generalized linear mixed model to examine temporal changes in alpha diversity and bacterial biomass. The same procedures and settings were applied as described in the section above.

References

1. Mollerup S, Worning P, Petersen A, Bartels MD. spa Typing of Methicillin-Resistant Staphylococcus aureus Based on Whole-Genome Sequencing: the Impact of the Assembler. Microbiol Spectr. 2022;10:e0218922. https://doi.org/10.1128/spectrum.02189-22
2. Liu CM, Erikstrup LT, Edslev SM, Park DE, Salazar JE, Aziz M, et al. Composition and dynamics of the adult nasal microbiome. Microbiome. 2026;14:38. https://doi.org/10.1186/s40168-025-02250-3
3. Fadrosh DW, Ma B, Gajer P, Sengamalay N, Ott S, Brotman RM, et al. An improved dual-indexing approach for multiplexed 16S rRNA gene sequencing on the Illumina MiSeq platform. Microbiome. 2014;2:6. https://doi.org/10.1186/2049-2618-2-6
4. Liu CM, Aziz M, Kachur S, Hsueh PR, Huang YT, Keim P, et al. BactQuant: an enhanced broad-coverage bacterial quantitative real-time PCR assay. BMC Microbiol. 2012;12:56. https://doi.org/10.1186/1471-2180-12-56
5. Iversen S, Johannesen TB, Ingham AC, Edslev SM, Tevell S, Månsson E, et al. Alteration of Bacterial Communities in Anterior Nares and Skin Sites of Patients Undergoing Arthroplasty Surgery: Analysis by 16S rRNA and Staphylococcal-Specific tuf Gene Sequencing. Microorganisms. 2020;8. https://doi.org/10.3390/microorganisms8121977
6. Martin M. Cutadapt removes adapter sequences from high-throughput sequencing reads. EMBnetjournal. 2011;17:10-2.
7. Callahan BJ, McMurdie PJ, Rosen MJ, Han AW, Johnson AJ, Holmes SP. DADA2: High-resolution sample inference from Illumina amplicon data. Nat Methods. 2016;13:581-3. https://doi.org/10.1038/nmeth.3869
8. McLaren MR, Callahan BJ. Silva 138.1 prokaryotic SSU taxonomic training data formatted for DADA2. Zenodo https://doiorg/105281/zenodo4587955. 2021.
9. McMurdie PJ, Holmes S. phyloseq: an R package for reproducible interactive analysis and graphics of microbiome census data. PLoS One. 2013;8:e61217. https://doi.org/10.1371/journal.pone.0061217
10. Wickham H. ggplot2: Elegant Graphics for Data Analysis. New York: Springer-Verlag; 2016.
11. Lê S, Josse J, Husson F. FactoMineR: An R Package for Multivariate Analysis. Journal of Statistical Software. 2008;25:1-18. https://doi.org/10.18637/jss.v025.i01
12. Kassambara A, Mundt F. Factoextra: Extract and Visualize the Results of Multivariate Data Analyses 2020 [Available from: https://CRAN.R-project.org/package=factoextra
13. Oksanen J, Simpson GL, Blanchet FG, Kindt R, Legendre P, Minchin PR, et al. vegan: Community Ecology Package 2022 [Available from: https://CRAN.R-project.org/package=vegan.
14. Mallick H, Rahnavard A, McIver LJ, Ma S, Zhang Y, Nguyen LH, et al. Multivariable association discovery in population-scale meta-omics studies. PLoS Comput Biol. 2021;17:e1009442. https://doi.org/10.1371/journal.pcbi.1009442
15. Bates B, Mächler M, Bolker B, Walker S. Fitting Linear Mixed-Effects Models Using lme4. Journal of Statistical Software. 2015;67:1-48. https://doi.org/10.18637/jss.v067.i01
