# Supplementary material for: Changes in nasal and throat microbiota composition during and after mupirocin and chlorhexidine decolonization treatment in asymptomatic methicillin-resistant Staphylococcus aureus carriers: a longitudinal observational study
Source: Clin Microbiol Infect. Author manuscript; Available in PMC 2026 Sep 12. (PMC13570456; doi:10.1016/j.cmi.2026.02.020)
Supplement: Supplementary file 2 [file NIHMS2190782-supplement-2.pdf]

Supplemental tables and figures

Table S1. Distribution of MRSA sequence types (ST) stratified by decolonization outcome

ST	CC	Unsuccessful decolonisation (n=13)	Successful decolonisation (n=21)
ST1	CC1	n=2 (15%)	n=1 (4.8%)
ST5	CC5	n=2 (15%)	n=1 (4.8%)
ST6	CC6	n=2 (15%)	n=1 (4.8%)
ST1649	CC6	n=2 (15%)	n=1 (4.8%)
ST4110	CC6	n=1 (7.7%)	n=0
ST8	CC8	n=1 (7.7%)	n=2 (9.5%)
ST1535	CC15	n=0	n=1 (4.8%)
ST22	CC22	n=1 (7.7%)	n=3 (14%)
ST30	CC30	n=0	n=4 (19%)
ST59	CC59	n=1 (7.7%)	n=2 (9.5%)
ST88	CC88	n=1 (7.7%)	n=0
ST97	CC97	n=0	n=1 (4.8%)
ST3077	CC97	n=0	n=1 (4.8%)
ST1413	CC121	n=0	n=1 (4.8%)
ST188	CC188	n=0	n=1 (4.8%)
ST672	-	n=0	n=1 (4.8%)

Sequence types (STs) were determined based on whole-genome sequencing of MRSA strains isolated prior to the study recruitment. Clonal complex (CC) type was determined using PubMLST (https://pubmlst.org/bigsdb?db=pubmlst_saureus_seqdef&page=profiles). Decolonisation outcome was assessed at the 30-day clinical follow-up by culturing swabs from the nose, throat, and perineum, with a MRSA-positive sample from any tested body site indicating unsuccessful decolonisation.

Table S2. Prevalence of nasal CST in MRSA carriers at baseline compared to the reference group

**Nasal CSTs**	**MRSA-carriers (N=34) % (95% CI)**	**Reference group (N=116) % (95% CI)**	**P-value**	**FDR adj. p-value**	***S. aureus* carriers in the reference group (N=38) % (95% CI)**	**P-value**	**FDR adj. p-value**
S. aureus	201% (8.7-38%)	4.3% (1.4-9.8%)	0.006	**0.02**	13% (4.4-28%)	0.5	0.7
CoNS	24% (12-41%)	1.7% (0.2-6.1%)	0.0001	**0.0008**	2.6% (0.1-14%)	0.01	0.08
Dolosigranulum	5.9% (0.7-20%)	17% (11-25%)	0.2	0.3	16% (6-31%)	0.3	0.7
Moraxella	8.8% (1.9-24%)	11% (6.1-18%)	1.0	1.0	5.3% (0.6-18%)	0.7	0.7
Haemophilus	2.9% (0.1-15%)	1.7% (0.2-6.1%)	0.5	0.7	0	0.5	0.7
Corynebacterium	18% (6.8-35%)	44% (35-54%)	0.005	**0.02**	40% (24-57%)	0.07	0.3
Cutibacterium	18% (6.8-35%)	19% (12-27%)	1	1	24% (11-40%)	0.6	0.7
Other	2.9% (0.1-15%)	0.9% (0%-4.7%)	0.4	0.6	0	0.5	0.7

Pairwise comparisons of CST distributions in MRSA carriers at baseline with the reference group (test 1) and with the subset of *S. aureus* carriers in the reference group (test 2) were performed using Fisher's exact test, with p-values adjusted for multiple testing using the Benjamini–Hochberg method. Abbreviations: CST: Community state types. CoNS: Coagulase-negative Staphylococcus. CI: Confidence levels. FDR: False discovery rate.

Table S3. Bacterial taxa abundances at baseline as predictors of nasal MRSA decolonization

Taxa	Multivariate analysis			Univariate analysis		
	Estimate (95%CI)	P-value	FDR adj. p-value	Estimate (95%CI)	P-value	FDR adj. p-value
Acinetobacter	12 (-41;103)	0.7	0.8	16 (-32;102)	0.6	0.9
Anaerococcus	4.0 (-20;39)	0.8	0.8	1.9 (-22;36)	0.9	0.9
Campylobacter	6.9 (-26;76)	0.7	0.8	6.2 (-26;74)	0.8	0.9
Corynebacterium	-2.6 (-8.2;2.7)	0.3	0.8	-3.0 (-8.3;2.2)	0.3	0.9
Cutibacterium	2.3 (-2.9-11)	0.5	0.8	2.4 (-2.3;10)	0.4	0.9
Dolosigranulum	5.5 (-17;40)	0.7	0.8	3.2 (-19;37)	0.8	0.9
Finegoldia	21 (-22;87)	0.4	0.8	18 (-23;80)	0.5	0.9
Haemophilus	-11 (-40;19)	0.4	0.8	-13 (-41;17)	0.3	0.9
Lawsonella	-4.3 (-25;20)	0.7	0.8	-4.5 (-26;20)	0.7	0.9
Peptoniphilus	38 (1.8;102)	0.1	0.8	32 (-0.4;91)	0.2	0.9
Prevotella	39 (-34;163)	0.4	0.8	32 (-39;153)	0.5	0.9
CoNS	1.5 (-3.6;8.8)	0.6	0.8	1.7 (-3.3;9.1)	0.6	0.9
S. aureus	-1.8 (-6.9;3.5)	0.5	0.8	-1.4 (-5.9;3.6)	0.5	0.9
Streptococcus	1.0 (-5.5;17)	0.8	0.8	0.9 (-5.6;18)	0.8	0.9
Neisseriaceae	1.4 (-8.7;17)	0.8	0.8	0.5 (-9.4;15)	0.9	0.9

The table presents results from logistic regression models testing whether the relative abundance of nasal bacterial taxa at baseline (explanatory variable) was associated with nasal MRSA decolonization (response variable; 1: successful nasal decolonization, 0: persistent nasal colonization). Each taxon was tested individually, and p-values were adjusted for multiple testing using the Benjamini–Hochberg method. Both univariate models and multivariate models, including MRSA throat carriage at baseline, were performed. Any taxon present in >15% of the samples and with a relative abundance >1% in a minimum 15% of samples was tested. Abbreviations: CoNS: Coagulase-negative *Staphylococcus*. CI: Confidence levels. FDR: False discovery rate.

Table S4. Bacterial taxa colonisation at baseline as predictors of nasal MRSA decolonization

	Multivariate analysis			Univariate analysis		
**Taxa**	**Estimate (95%CI)**	**P-value**	**FDR adj. p-value**	**Estimate (95%CI)**	**P-value**	**FDR adj. p-value**
Acinetobacter	0.8 (-1.3;2.9)	0.4	0.9	0.8 (-1.3;2.8)	0.4	0.9
Anaerococcus	-0.2 (-3.3;2.0)	0.9	0.9	-0.3 (-3.4;1.8)	0.8	0.9
Campylobacter	-0.5 (-2.3;1.3)	0.6	0.9	-0.6 (-2.3;1.2)	0.5	0.9
Dolosigranulum	0.5 (-1.2;2.6)	0.6	0.9	0.4 (-1.3;2.5)	0.7	0.9
Haemophilus	0.6 (-1.2;2.4)	0.5	0.9	0.8 (-1.0;2.5)	0.4	0.9
Lawsonella	-1.1 (-4.1;0.9)	0.3	0.9	-1.1 (-4.1;0.9)	0.3	0.9
Peptoniphilus	0.5 (-1.3;2.3)	0.6	0.9	0.4 (-1.4;2.1)	0.6	0.9
Prevotella	0.3 (-1.5;2.1)	0.8	0.9	0.1 (-1.6;1.8)	0.9	0.9
S. aureus	-1.1 (-4.1;0.9)	0.3	0.9	-0.9 (-3.9;1.1)	0.4	0.9

The table presents results from logistic regression models testing whether colonization of nasal bacterial taxa at baseline (explanatory variable) was associated with nasal MRSA decolonization (response variable; 1: successful nasal decolonization, 0: persistent nasal colonization). Each taxon was tested individually, and p-values were adjusted for multiple testing using the Benjamini–Hochberg method. Both univariate models and multivariate models, including MRSA throat carriage at baseline, were performed. Taxon colonization was defined as a relative abundance >0.1%, and any taxon present in <85% of the samples or present >15% of the samples was tested. Abbreviations: CoNS: Coagulase-negative *Staphylococcus*. CI: Confidence levels. FDR: False discovery rate.

Table S5. Results from differential abundance analysis comparing the abundance of bacterial taxa in MRSA carriers at day 2 and day 30 compared to baseline (day 0)

Bacterial taxa	(Day 2 vs. Day 0)			(Day 30 vs. Day 0)		
	**Log_2 fold change**	**P-value**	**FDR adj. p-value**	**Log_2 fold change**	**P-value**	**FDR adj. p-value**
S. aureus	-2.2	0.0001	**0.002**	-3.6	<0.0001	**0.001**
CoNS	-1.9	<0.0001	**<0.0001**	-1.1	0.03	0.09
Micrococcus	1.3	0.003	**0.02**	0.6	0.09	0.2
Dolosigranulum	-1.2	0.03	0.1	-1.5	0.09	0.2
Paracoccus	1.0	0.03	0.1	nt	nt	nt
Rothia	1.0	0.03	0.1	1.2	0.004	**0.02**
Cutibacterium	0.5	0.05	0.1	0.6	0.04	0.1
Streptococcus	-0.5	0.3	0.5	0.7	0.02	0.09
Corynebacterium	-0.3	0.1	0.3	1.9	<0.0001	**<0.0001**

The table contains all results from two Maaslin2 models assessing taxa being differentially abundant in MRSA carriers at day 2 (second day of treatment) and day 30 as compared to baseline (day 0). taxa with a p-value <0.05 in either model are included in the table. A total of 21 and 19 taxa were tested in each model (i.e., taxa present in a minimum of 15% of the samples and with a relative abundance >1% in a minimum of 15% of the samples). A Log_2 fold change >0 indicate that the bacteria is increased in the follow-up samples (day 2 and day 30), compared to baseline, whereas a Log_2 fold-change <0 indicate that the bacteria is decreased in the follow-up samples. The table is sorted based on the taxa showing the greatest difference between time points in the first model. nt: not tested.

Table S6: Results from differential abundance analysis comparing the abundance of bacterial taxa in MRSA carriers and the reference group

	MRSA carriers (Day 0) vs. the reference group			MRSA carriers (Day 30) vs. the reference group			MRSA carriers (Day 90) vs. the reference group			MRSA carriers (Day 180) vs. the reference group		
**Bacterial taxa**	**Log_2 fold change**	**P-value**	**FDR adj. p-value**	**Log_2 fold change**	**P-value**	**FDR adj. p-value**	**Log_2 fold change**	**P-value**	**FDR adj. p-value**	**Log_2 fold change**	**P-value**	**FDR adj. p-value**
Acinetobacter	1.9	0.0002	**0.001**	2.1	<0.0001	**<0.001**	nt	nt	nt	nt	nt	nt
CoNS	-0.1	0.7	0.9	-1.3	0.002	**0.01**	-0.02	1	1	0.2	0.8	1
Corynebacterium	-1.8	<0.0001	**<0.0001**	0.2	0.5	0.6	-0.2	0.6	0.8	0.08	0.8	1
Cutibacterium	-0.9	0.002	**0.006**	-0.3	0.3	0.4	-0.9	0.05	0.2	-1.2	0.007	**0.05**
Dolosigranulum	-2.6	0.004	**0.01**	-4.2	<0.0001	**<0.0001**	-5.4	<0.0001	**<0.001**	-4.7	<0.001	**0.006**
Lawsonella	-2.2	0.002	**0.006**	-1.6	0.02	0.06	-2.8	0.005	**0.04**	-2.3	0.03	0.1
Moraxella	-1.7	0.08	0.1	-3.0	<0.001	**0.005**	-2.9	0.04	0.2	-3.0	0.04	0.1
Peptoniphilus	-1.5	0.02	**0.05**	-1.4	0.02	0.07	-1.5	0.1	0.3	-1.0	0.3	0.6
S. auresu	3.2	<0.0001	**<0.0001**	-0.04	0.9	1	-1.0	0.2	0.4	-0.4	0.6	1

The table contains all significant results (adj. p-value <0.05) from a Maaslin2 analysis of taxa being differentially abundant in MRSA carriers at baseline (day 0) and post decolonization treatment (i.e. day 30, day 90, and day 180) compared to the reference population. All taxa present in a minimum of 15% of the samples and with a relative abundance >1% in a minimum of 15% of the samples, were included in the analysis (i.e. 16 taxa, 18 taxa, 14 taxa, and 14 taxa in the four tests respectively). nt, indicates that the taxa were not included in the test. A log_2 fold change >0 indicate that the bacteria is increased in MRSA carriers, whereas a log_2 fold change <0 indicate that the bacteria is decreased in MRSA carriers compared to the reference group. The table is sorted based on taxa names. Abbreviations: CoNS: Coagulase negative *Staphylococcus.*

Table S7. Results from differential abundance analysis comparing the abundance of bacterial taxa in the throat at day 7 compared to day 0 and day 30 respectively

Bacterial taxa	Day 0 (ref) vs. Day 7			Day 7 (ref) vs. Day 30		
	Log_2 fold change	P-value	FDR adj. p-value	Log_2 fold change	P-value	FDR adj. p-value
Actinomyces	0.1	0.01	**0.03**	-0.02	0.2	0.3
Campylobacter	-0.3	<0.0001	**<0.0001**	0.1	<0.0001	**0.0003**
Fusobacterium	-0.4	<0.0001	**<0.0001**	0.1	0.002	**0.01**
Johnsonella	nt	nt	nt	-0.1	0.006	**0.02**
Lachnoanaerobaculum	0.2	0.0004	**0.002**	-0.1	0.004	**0.01**
Leptotrichia	-0.5	<0.0001	**<0.0001**	0.1	<0.0001	**0.0003**
Neisseria	-0.5	<0.0001	**<0.0001**	0.1	<0.0001	**0.0009**
Oribacterium	0.1	0.1	0.2	-0.03	0.005	**0.02**
Rothia	0.2	0.006	**0.02**	-0.02	0.2	0.3
Selenomonas	0.1	0.01	**0.03**	-0.1	0.002	**0.01**
Stomatobaculum	0.2	0.003	**0.01**	-0.04	0.02	0.053
Streptococcus	-0.1	0.007	**0.02**	0.04	0.003	**0.01**

The table contains all significant results (adj. p-value < 0.05) from Maaslin2 analysis of taxa being differentially abundant in the throat at day 7 compared to day 0 (test 1) and day 30 (test 2). A total of 21 taxa were included in test 1 and 25 taxa were included in test 2 (i.e., taxa present in a minimum of 15% of the samples and with a relative abundance >1% in a minimum of 15% of the samples). nt indicates that the taxa was not included in the specific test. Of notice, the relative abundance of *S. aureus* was < 1% at any given time point and therefor not tested. A log2 fold change > 0 indicate that the bacteria is increased compared to the reference time point, whereas a log2 fold-change < 0 indicate that the bacteria is decreased.

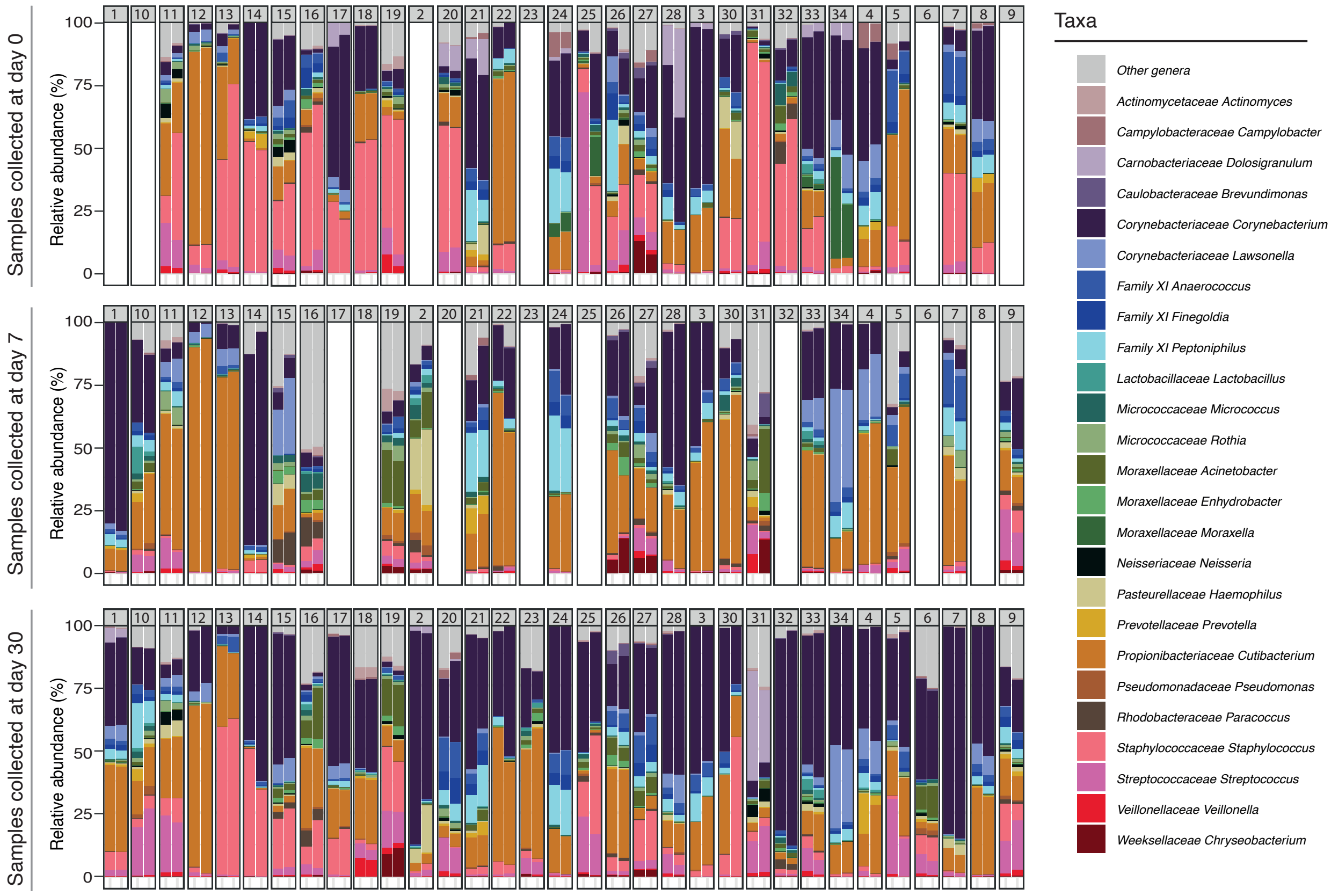

Figure S1. Comparison of paired nasal samples collected by participants and a professional. During the study follow-up, participants collected nasal swabs themselves. To validate the quality of self-sampling in nasal microbiome studies, paired samples were also collected by a trained medical student at three study visits (day 0, day 7, and day 30). After quality filtering, including removal of contaminants and exclusion of samples with fewer than 2,500 sequences, a total of 85 paired samples (n=170) were included in the comparative analysis. Taxonomic composition within paired samples was compared by visual inspection of taxa bar plots (shown above) and by hierarchical clustering based on Bray–Curtis distances at the ASV level (dendrogram not shown). The taxa bar plots revealed similar distributions of the 25 most abundant genera, and the dendrogram indicated tight clustering of the paired samples. Sequence depth was also similar between samples collected by professionals (median sequence depth: 8,828 reads, IQR:6,235-15,772) and participants (median sequence depth: 11,151 reads, IQR:5,748-16,130) ($p = 0.6$). Together, these results indicate that self-sampling for nasal microbiome analysis yields data of comparable quality to that obtained from samples collected by professionals. Abbreviations: ASV: amplicon sequence variants; IQR: interquartile range.

A

ASVs	NCBI BLAST results	Results based on *tuf* data
ASV2	*S. epidermidis*	*S. epidermidis*
ASV16	*S. aureus*	*S. aureus*
ASV24	*S. capitis*	*S. capitis*
ASV29	*S. epidermidis* + uncultured bacteria	*S. epidermidis*
ASV52	*S. lugdunensis*	*S. lugdunensis*
ASV64	*S. epidermidis* + uncultured bacteria	*S. epidermidis*
ASV67	*S. hominis*	*S. hominis*
ASV156	*S. epidermidis*	*S. epidermidis*

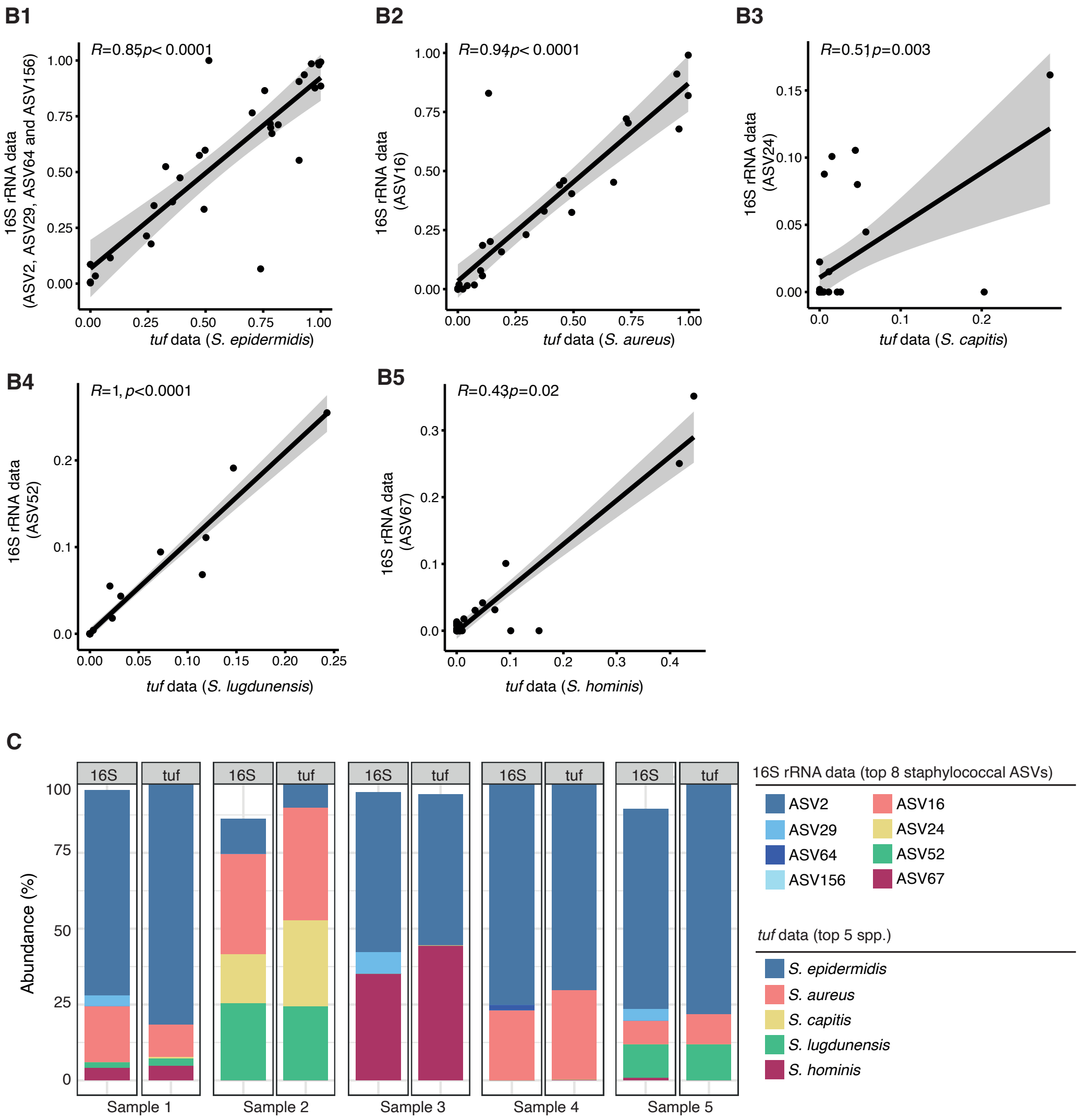

Figure S2. Staphylococcal species classification. ASVs derived from staphylococcal genomes were classified as *S. aureus* or coagulase-negative *Staphylococcus* (CoNS) using the following analytical approaches. First, the eight most abundant staphylococcal ASVs were classified at the species level using (i) BLAST searches against the nucleotide reference database maintained by the National Center for Biotechnology Information (NCBI) (**A**), and (ii) comparison with staphylococcal species data obtained from amplicon sequencing of the *tuf* gene in a subset of samples (n=31) (**A, B, C**). Spearman correlation analysis was used to compare the relative abundance of these eight ASVs in the 16S rRNA dataset with the relative abundance of the five most abundant staphylococcal species in the *tuf* dataset within each sample (**B**). **C**) include sample-wise comparisons of the relative abundance of staphylococcal ASVs (top eight) in the 16S rRNA data and relative abundance of species (top five) in the *tuf* dataset in five selected samples. Few bars are

not filled completely indicating that other staphylococcal ASVs and/or species are present in the sample. Combined, these analyses indicated that ASV2, ASV29, ASV64, and ASV156 most likely derived from *S. epidermidis*; ASV16 from *S. aureus*; ASV24 from *S. capitis*; ASV52 from *S. lugdunensis*, and ASV67 from *S. hominis*. These eight ASVs accounted for the majority (90.1%) of sequence counts among all staphylococcal ASVs (N=205) in the 16S rRNA dataset, making their classification a robust starting point. Next, an alignment of all staphylococcal ASV sequences was used to build an SNP-based phylogeny (not shown). ASVs clustering with ASV16 were inferred to originate from *S. aureus*, whereas those clustering with ASV2, ASV29, ASV64, ASV156, ASV24, ASV52, or ASV67 were inferred to derive from CoNS species. Furthermore, investigation of sequence diversity in the V3–V4 region of the 16S rRNA gene across staphylococcal species have previous showed that *S. aureus* can be distinguished from CoNS species by ≥3 SNPs (Iversen et al. 2020, doi: 10.3390/microorganisms8121977). In contrast, no SNP differences were observed between *S. aureus* and the two other coagulase-positive species, *S. argenteus* and *S. schweitzeri*, in the V3–V4 region. However, these three species can be distinguished using the *tuf* sequencing method, and analysis of the *tuf* data showed that none of the 31 samples contained *S. schweitzeri*, while *S. argenteus* was only detected in one nasal sample, where it represented 0.07% of the total staphylococcal abundance in that sample. Thus, it is highly unlikely that a significant proportion of *S. aureus* ASVs in this study were misclassified and actually derived from other coagulase-positive staphylococci.

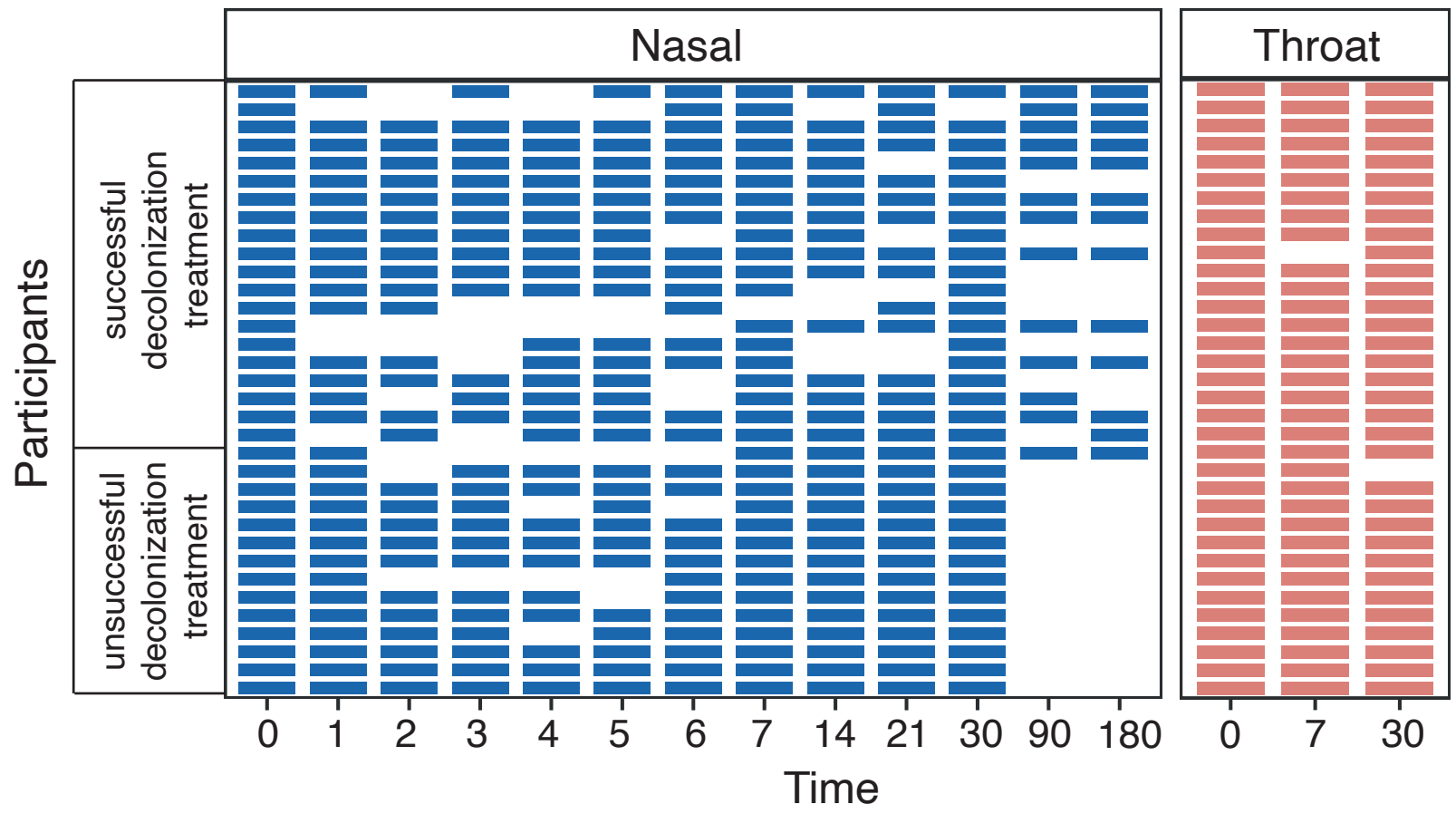

Figure S3. Overview of samples included in the study. Each row represents a participant, and a coloured tile indicates that a sample from the specified time point was included in the study. Late follow-up samples (day 90 and day 180) were not collected from participants with an unsuccessful treatment, since these individuals likely initiated secondary treatment. Decolonisation outcome was assessed at the 30-day clinical follow-up by culturing swabs from the nose, throat, and perineum, with a MRSA-positive sample from any tested body site indicating unsuccessful decolonisation. Missing samples were due to i) loss to follow-up among participants with successful decolonization, ii) missing labels on received samples, or iii) exclusion due to low sequence depth.

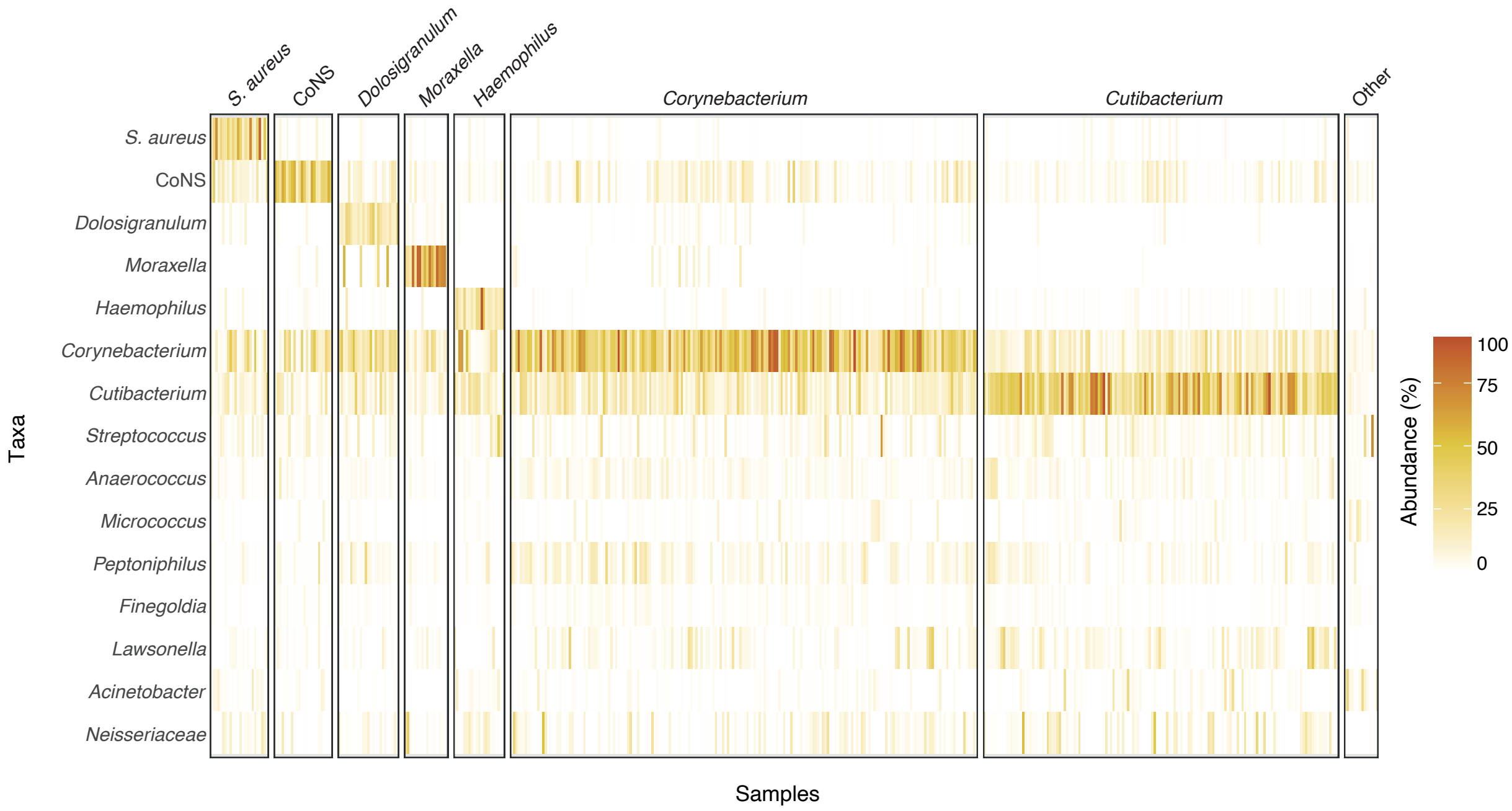

Figure S4. Bacterial community types. The plot shows the relative abundance of the 15 most abundant taxa in all the nasal samples (x-axis). Samples are grouped into bacterial community groups based on the relative abundance of seven key taxa in the nasal cavity, i.e., *S. aureus*, CoNS, *Dolosigranulum*, *Moraxella*, *Haemophilus*, *Corynebacterium*, and *Cutibacterium*. The assignment was based on the following ordered criteria: 1) samples with a relative abundance of *S. aureus* >10% were assigned to the *S. aureus* group; 2) samples with a relative abundance of *Dolosigranulum* >10% were assigned to *Dolosigranulum* group, 3) samples with a relative abundance of *Haemophilus* >10% were assigned to *Haemophilus* group , 4) samples with a relative abundance >10% of CoNS, *Moraxella*, *Corynebacterium*, and/or *Cutibacterium* were assigned to the corresponding group and in cases where the proportional abundance were > 10% for several of these taxa, the assignment was based on the taxa with the highest relative abundance. In a few samples, none of these taxa were present at higher abundance (>10%), and these samples were grouped as "other taxa". Abbreviations: CoNS: Coagulase-negative *Staphylococcus.*

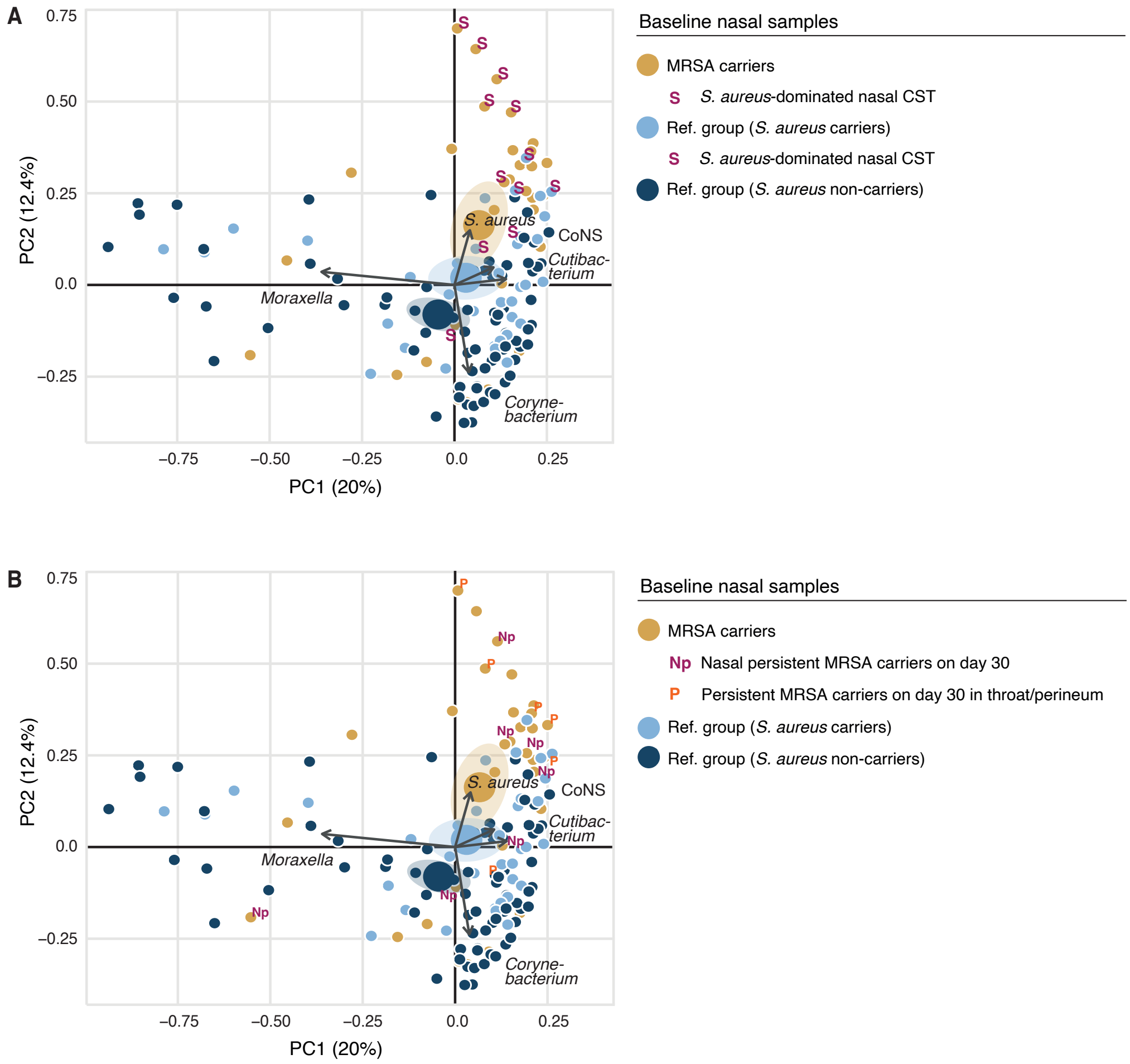

Figure S5. PCA biplot comparing nasal bacterial composition in MRSA carriers at baseline with the reference group, stratified by *S. aureus* carriage. The PCA biplot corresponds to Figure 2D but provides additional information on **(A)** clustering of samples assigned to the *S. aureus*–dominated CST and **(B)** clustering of samples collected from participants who remained colonised with MRSA at the 30-day clinical follow-up (unsuccessful decolonisation). Np: participants who tested MRSA-positive in the nose, and possibly also in the throat or perineum. P: participants who were MRSA-negative in the nose but MRSA-positive in the throat and/or perineum.

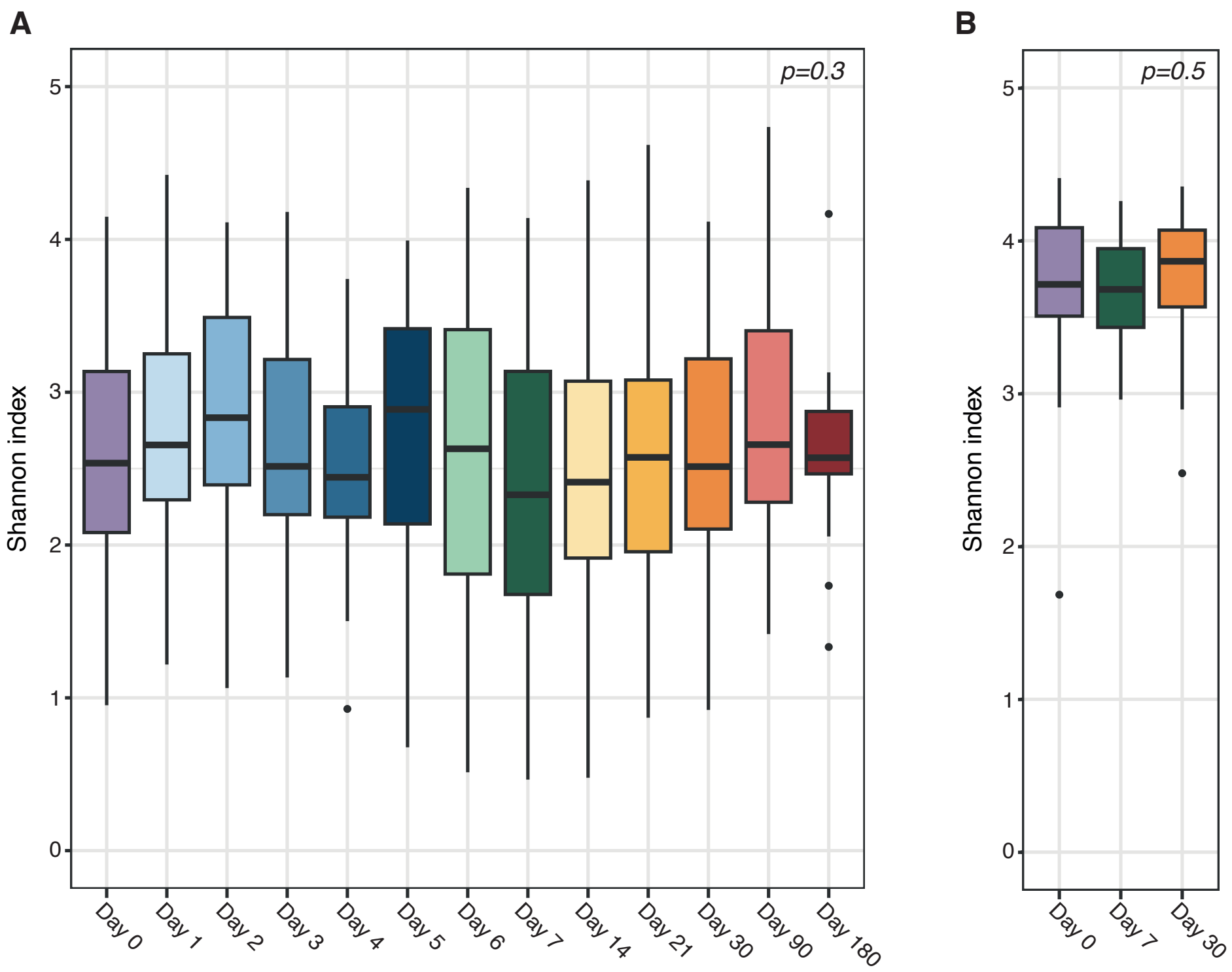

Figure S6. Alpha diversity. Box plots show the alpha diversity within the nasal (**A**) and throat (**B**) microbiota, measured by the Shannon index at ASV level. Significance was assessed using generalized mixed models, with participant ID included as a random factor. Nasal samples collected at day 90 and day 180 were excluded from the statistical testing. Abbreviations: ASV: amplicon sequence variants.

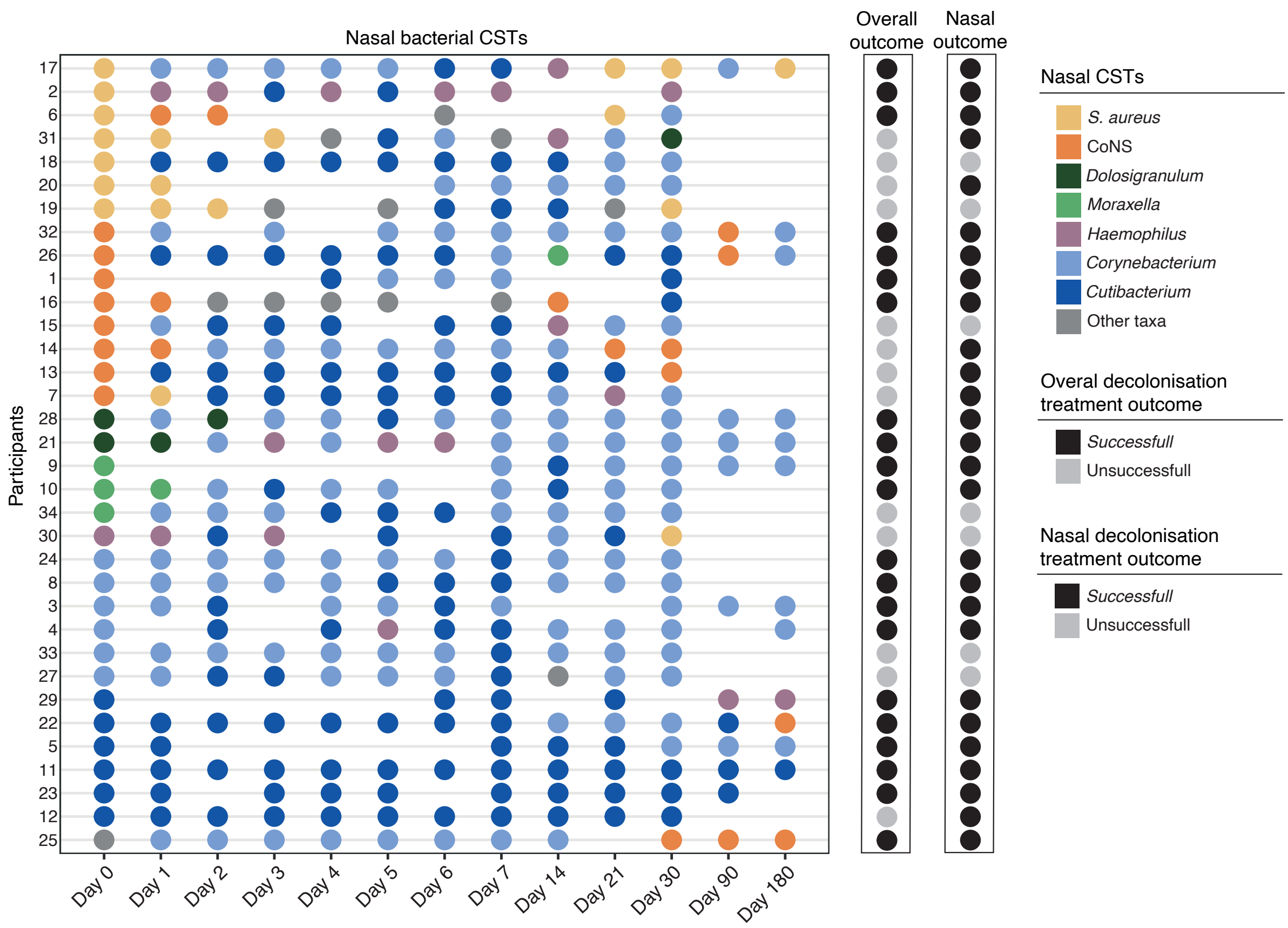

Figure S7. Temporal changes in nasal bacterial community profiles. The dot plot shows changes in bacterial nasal community types (CSTs) within participants (y-axis) over time (x-axis). Participants are sorted based on CSTs at baseline. The column "Overall outcome" indicates whether participants had a successful or unsuccessful MRSA decolonization at all tested body sites, whereas "Nasal outcome", indicates if the participants were MRSA-positive (unsuccessful decolonisation) or MRSA-negative (successful decolonisation) in the nose at the 30-day clinical follow-up. Abbreviations: CoNS: coagulase-negative *Staphylococcus*.

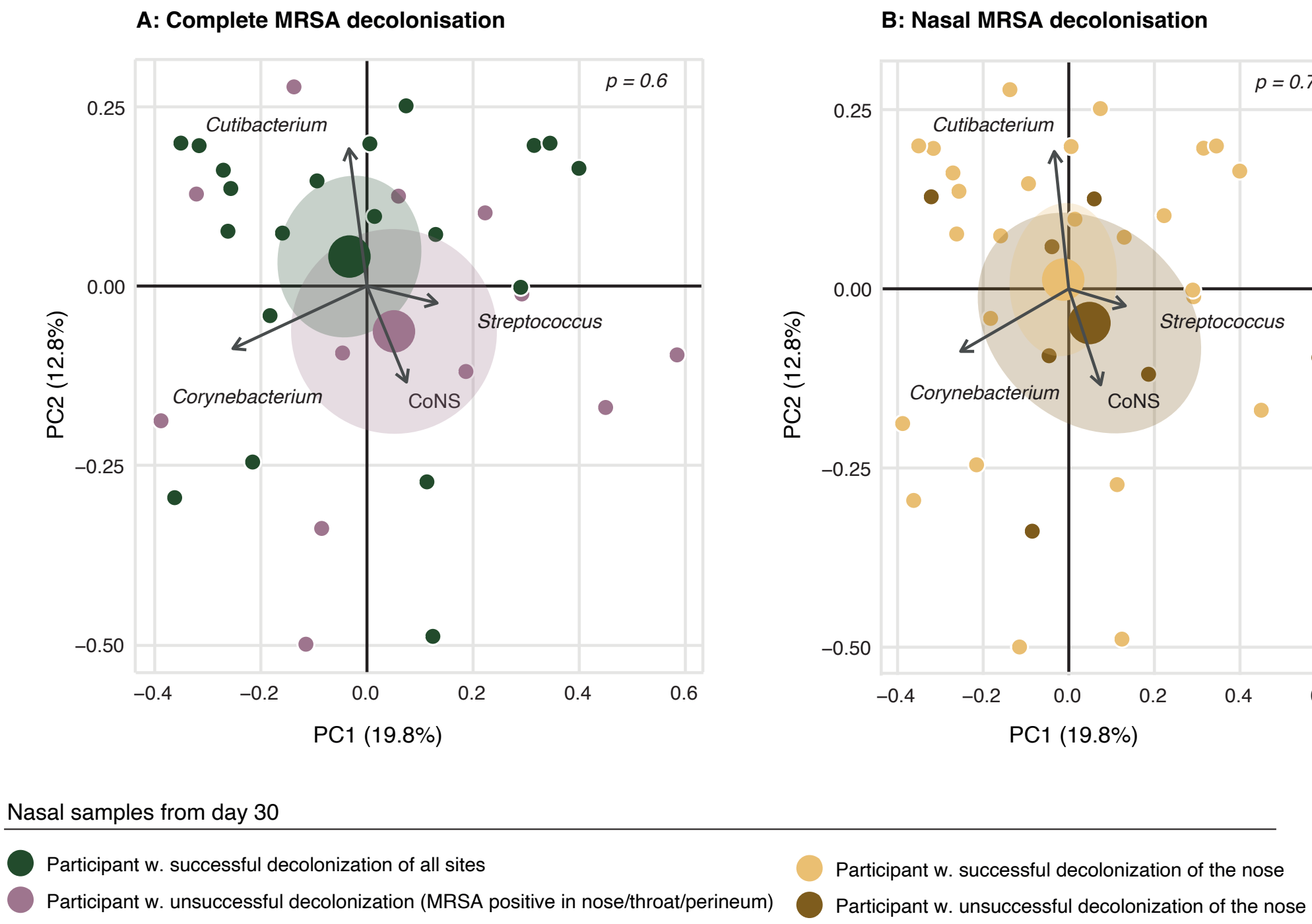

Figure S8. Comparison of the nasal community composition post-decolonization treatment between participants with successful and unsuccessful decolonization. PCA biplot comparing nasal bacterial communities after treatment (day 30) in participants with successful versus unsuccessful decolonization, where successful decolonisation defined as **(A)** MRSA-negative at all tested body sites (nose, throat, and perineum), or **(B)**, MRSA-negative in the nose (but possibly tested positive at other body sites). *Corynebacterium* contributed most to the variation along PC1 (49.5%), while *Cutibacterium* (44.0%) and CoNS (21.5%) contributed most to the variation along PC2. Larger points represent the mean for each sampling time point, with 95% confidence intervals shown as ellipses.

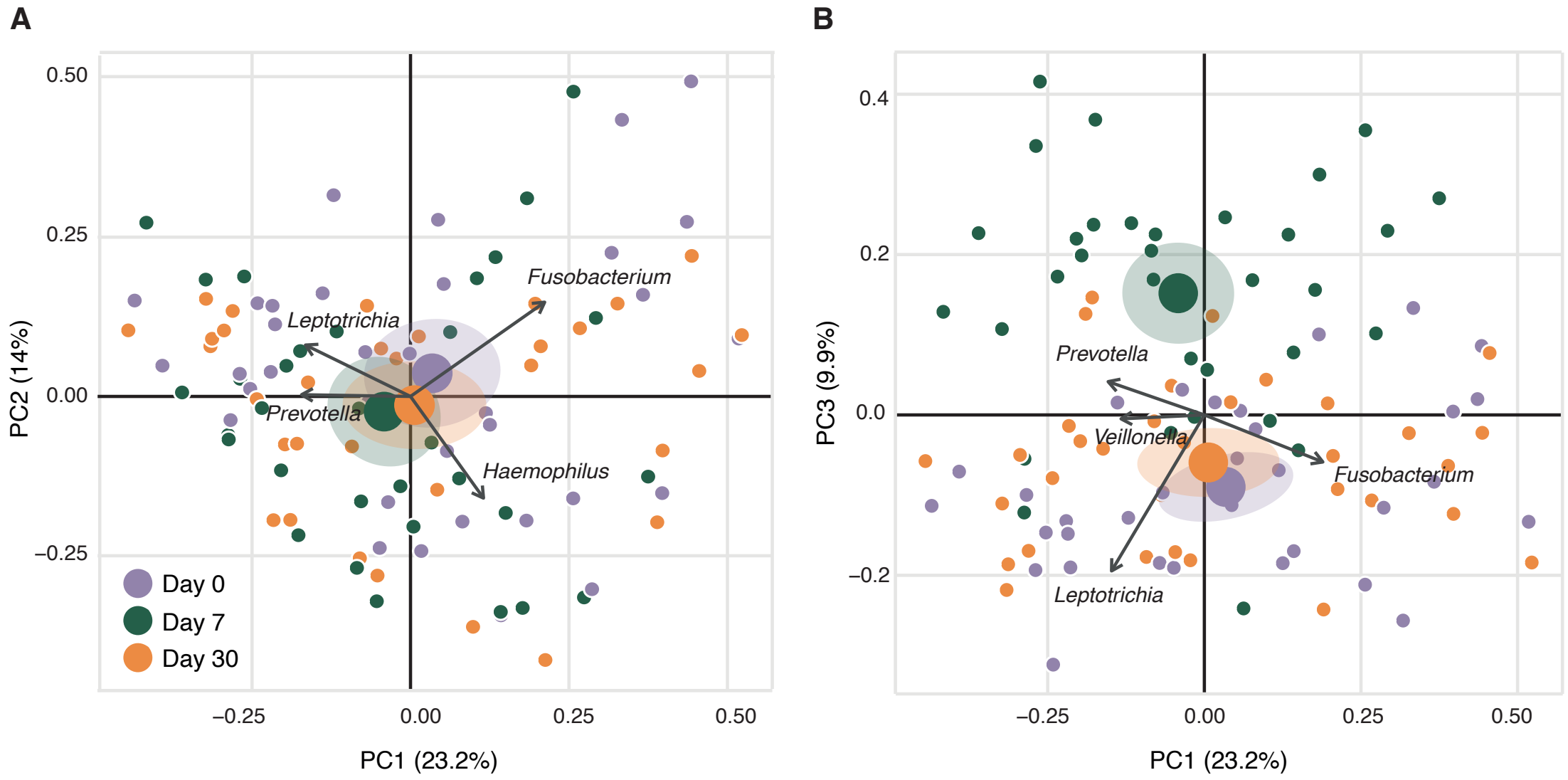

Figure S9. Temporal dynamics of the throat microbiota. The PCA-biplots illustrate bacterial compositional variations in the throat between sampling time points. **(A)** shows sample distributions along PC1 and PC2, and **(B)** along PC1 and PC3. The plots indicate that samples collected at day 7 (green) were separated from samples collected at day 0 (purple) and day 30 (orange) along PC3, which explained 9.9% of the total variation. *Leptotrichia* contributed most to the variation within PC3 (49.2%). Abbreviations: PCA: principal component analysis.
